# Multi-omic analysis of PBMCs in sepsis reveals widespread cytotoxic dysfunction and an increased population of *CD69* expressing naïve CD4+ T cells

**DOI:** 10.3389/fimmu.2025.1667186

**Published:** 2025-10-24

**Authors:** Joshua Flynn, Anne Marie Baird, Eamon Breen, Michael Carty, Ciara S. McNevin, Lisa McDermott, Elaine M. Kenny, John Davis Coakley, Thomas Ryan, Derek G. Doherty, Orla Sheils

**Affiliations:** ^1^ School of Medicine, Trinity Translational Medicine Institute, Trinity College Dublin, Dublin, Ireland; ^2^ Trinity St James’s Cancer Institute, Dublin, Ireland; ^3^ Department of Histopathology and Morbid Anatomy, Trinity Translational Medicine Institute, Trinity College Dublin, Dublin, Ireland; ^4^ Flow Cytometry Facility, Trinity Translational Medicine Institute, Trinity College Dublin, Dublin, Ireland; ^5^ Department of Clinical Microbiology, Trinity Translational Medicine Institute, Trinity College Dublin, Dublin, Ireland; ^6^ TrinSeq, Trinity Translational Medicine Institute, Trinity College Dublin, Dublin, Ireland; ^7^ Department of Intensive Care Medicine, St James’s Hospital, Dublin, Ireland; ^8^ Department of Immunology, Trinity Translational Medicine Institute, Trinity College Dublin, Dublin, Ireland

**Keywords:** sepsis, multi-omic, single-cell sequencing, immunosuppression, cytotoxicity, inflammation

## Abstract

**Introduction:**

Sepsis is responsible for 1 in 5 deaths globally and the majority of those who survive have lasting health issues. A hallmark of sepsis is a deregulated inflammatory response to infection, with leukocytes playing a critical role.

**Methods:**

This study utilised a targeted single-cell multi-omics approach to characterise peripheral blood mononuclear cell (PBMC) populations and their transcriptomic profiles in an Irish cohort of people with (i) sepsis and (ii) bacteraemia without sepsis (defined as clinically significant positive blood culture without sepsis as assessed by the Clinical Microbiology service).

**Results:**

Variable leukocyte distributions were identified, with decreased cytotoxic lymphocytes, including CD8+ T cells, natural killer cells, CD56+ T cells, γδ T cells, mucosal-associated invariant T cells, and increased T helper (Th) cell subsets within sepsis samples. Additionally, PBMCs from sepsis samples displayed an impaired expression profile in effector T cells, resulting in widespread suppression of PBMC cytotoxic activity.

**Discussion:**

These results identify potential mechanisms underlying the functional impairment witnessed in sepsis. Such mechanisms may inform future diagnostic and treatment strategies.

## 1 Introduction

Sepsis is a life-threatening condition characterised by organ dysfunction associated with an aberrant host immune response to infection or trauma (1). Globally, the incidence of sepsis is estimated at over 48 million cases, with sepsis-related mortality reaching 11 million in 2017 (2). Several studies have highlighted the impact of the immune system in sepsis throughout the disease course (3–5). During infection, innate immune cells recognise pathogen-associated molecular patterns (PAMPs) and damage-associated molecular patterns (DAMPs), resulting in the production of inflammatory mediators to mount an effective immune response (6). However, these responses are exaggerated during sepsis, causing hyperinflammation and consequently damage to host tissue and organs (7). A prolonged period of immunosuppression typically follows which is characterised by increased expression of inhibitory and apoptotic markers on T cell populations (8–10).

Immune alterations may persist after recovery from sepsis, resulting in increased susceptibility to secondary infection and re-hospitalisation (11). Outcomes for these individuals are poor, with a significant proportion experiencing long-term sequelae, including cognitive impairment, cardiovascular complications, and increased risk from other comorbidities (12, 13).

The progression of sepsis is associated with perturbations in peripheral blood mononuclear cells (PBMCs) and their functions (10). Reprogramming of the innate immune system, such as increased activation of macrophages and NK cells may contribute to the cytokine storm characteristic of the sepsis clinical course (14, 15).The initial hyperinflammatory phase of sepsis is followed by subsequent decreases in effector T and B cells (4, 16, 17). Single-cell RNA sequencing (scRNA-seq) technology has facilitated the exploration of the transcriptional profiles of PBMCs, such as the characterisation of immune alterations in myeloid and lymphoid cells (18–21). Through scRNA-seq, new immune cell subsets are being defined (21). Furthermore, scRNA-seq studies have highlighted the marked immune cell heterogeneity displayed within sepsis-related PBMC populations (22, 23).

While our understanding of the pathophysiology of sepsis has improved in recent years, little progress has been made in improving treatment options (24). Understanding the disease aetiology is crucial to identifying novel prognostic markers and molecular and cellular targets for therapeutic intervention. In this current study, a novel targeted multi-omic approach using simultaneous measurement of immune cell transcriptome and proteome was performed. This approach concentrated on examining T and B lymphocyte markers in PBMCs from (i) patients with sepsis, (ii) those with bacteraemia without sepsis (Defined as individuals with a clinically significant positive blood culture indicating infection, but without sepsis as assessed by the Clinical Microbiology service. In line with Sepsis-3 criteria, these patients had SOFA scores <2 at the time of sampling), and (iii) healthy controls (25, 26). This integrative, multi-modal approach has identified specific populations of PBMCs, which are proportionally and functionally altered in those with sepsis.

## 2 Materials and methods

### 2.1 Study population

The project consisted of a pilot and study phase using the BD Rhapsody™ single-cell sequencing pipeline. This study utilised retrospective PBMC fractions isolated from patients with sepsis (n=14), bacteraemia (n=3) and healthy controls (n=3). The sepsis and bacteraemia samples were taken from patients in St. James’s Hospital ICU diagnosed according to the Sepsis 3 definition (**Supplementary Table S1**) (1). The bacteraemia samples were determined by the Clinical Microbiology service in St. James’s Hospital based on the presence of infection, identified through a significant positive blood culture, but without sepsis as based on a SOFA score < 2 at the time of blood sampling. The healthy control group was recruited from those attending a hospital phlebotomy service for community-based family practice and did not have any current or recent infections. The donors did not have any infective symptoms in the previous 8 weeks. Exclusion criteria included pre-existing immunodeficiency, immune modulating medications including steroids, chronic infection, malignancy, pre-existing liver disease, and any haematological disease. Further information on this cohort can be found in previously published studies (25, 26).

Ethical approval for this study, including sepsis, bacteraemia, and control samples, was granted by the St. James’s Hospital Research and Ethics Committee (Ethic approval code 2015-03).

### 2.2 BD Rhapsody™ pipeline

#### 2.2.1 Single cell labelling with the BD™ single-cell multiplexing kit and BD™ Abseq Ab-Oligos

PBMCs were isolated and processed using standard protocols. Alterations were made to the BD Rhapsody™ manufacturer’s protocol to increase PBMC viability and enhance capture rate. Briefly, the cells were blocked with an Fc Block solution (Stain Buffer (2% heat inactivated FBS (Merck) and 1 mM EDTA in PBS), and Fc Block (BD Pharmingen)), labelled with 2X AbSeq Ab-Oligos (**Supplementary Table S2**), Stain Buffer, and appropriate Sample Tag tube (BD Human Single-Cell Multiplexing Kit). Following incubation, each labelled cell suspension was centrifuged at 400 × g for 5 min, supernatant discarded, and pellet washed with 3 mL Stain Buffer and re-pelleted. This wash step was repeated 1–2 times. The final pellet was re-suspended in cold Sample Buffer (BD Rhapsody™ Cartridge Reagent Kit). Further information is provided in **Supplementary Information**.

#### 2.2.2 Single cell labelling, capture, and cDNA with the BD Rhapsody™ Single-cell analysis system

Due to decreased cell viability and low cell counts, modifications were made to the manufacturer’s protocol. The re-suspended cells were stained with 0.005 mM Draq7 (BD) and 0.01 mM Calcein AM (Thermo Fisher), incubated at 37 °C in the dark for 5 min, and filtered using a Cell Strainer Cap (Corning). In the initial workflow, stained suspensions were analysed directly on the BD Rhapsody™ scanner and loaded onto cartridges. However, due to the variability in cell health, the protocol was later adapted to incorporate a single viability sort on a BD FACS Melody. In the modified workflow, cells were first gated to exclude debris and doublets, and viability was assessed using Draq7 and Calcein AM, with live cells defined as Draq7^−^ Calcein AM^+^ and dead cells as Draq7^+^ Calcein AM^−^ (double-positive events excluded). Sorted live cells were collected into BD Rhapsody™ Sample Buffer prior to cartridge loading. The live cells were pelleted and re-suspended or sorted directly into cold Sample Buffer and pooled for preparation for loading onto the BD Rhapsody™ cartridge. The BD Rhapsody™ cartridge was primed, treated, and scanned according to manufacturers’ instructions. Cells were loaded onto the cartridge according to the manufacturer’s instructions, with the exception that initial incubation at room temperature was extended to 20 min. The Cell Capture Beads were prepared, loaded, and washed on the cartridge, followed by cell lysis and retrieval, and with final washing of Cell Capture Beads as per manufacturer’s instructions. Subsequently, the reverse transcription and exonuclease I treatment (BD Rhapsody™ cDNA Kit) was performed according to kit instructions. Further information is provided in the **Supplementary Information**.

#### 2.2.3 Library preparation

Three libraries were generated for each sample in this study (AbSeq, Sample Tag and mRNA). For mRNA libraries, the BD Rhapsody™ Immune Response Panel and a custom supplementary panel (**Supplementary Table S3**) were used. See **Supplementary Information** for further details.

#### 2.2.4 Sequencing

mRNA, Sample Tag and Abseq libraries were pooled (4 nM) diluted to 2.25-2.5 nM and spiked with 20% PhiX (Illumina). Sequencing runs were prepared and performed as per manufacturer’s instructions in the TrinSeq facility utilising v1.5 NovaSeq flowcells (Illumina; NovaSeq 6000 S1 Reagent Kit v1.5 (100 cycles)) on a NovaSeq 6000 (Illumina). See **Supplementary Information** for further details. Data from the sequencing runs were uploaded onto BaseSpace and converted to FASTQ files.

### 2.3 Quality control and data analysis

FASTQ files were uploaded to the Seven Bridges Genomics platform (Cambridge, MA, USA; https://www.sevenbridges.com) and processed through the BD Rhapsody™ pipeline (**Supplementary Information**) (27). Data is available under the accession PRJEB96265. RSEC-adjusted molecule counts, and expression matrices obtained from the BD Rhapsody™ pipeline were imported to RStudio for subsequent analysis using the R package Seurat 4.0 (https://github.com/satijalab/Seurat). Cells with low numbers of mRNA (<32 RNA features) and protein (<10 Abseq (ADT) features), and cells with very low mRNA (<100 RNA molecules) and protein (<1000 protein molecules) or very high levels of mRNA (>approx. 3000 RNA molecules) and protein (>40000 protein molecules) were filtered out. The resulting matrices were used for subsequent analyses.

### 2.4 Data processing

Data processing was performed using Seurat V4 (28). Individual mRNA and protein data from each sample was normalised using SCTransform (28) and centred log-ratio (CLR) methods (29), respectively. Protein and mRNA data were integrated separately using reciprocal principal component analysis (RPCA) for RNA and ADT, respectively (28). Following integration, dimensionality reduction and clustering was performed using the weighted nearest neighbours (WNN) protocol (28). For T cell analysis, contaminating B cells and clusters believed to be apoptotic were excluded. These were identified by contradictory lineage marker expression, high expression of all Abseq markers and low RNA content, or aberrant expression patterns (i.e., B cells expressing CD4 molecules). A cutoff of < 30, 000 Abseq counts per cell was then applied (see **Supplementary Information**).

### 2.5 Data analysis

Differential expression was performed using the FindMarkers function and accounting nCount_RNA using the MAST package for R (30). Cell type proportions for sepsis, bacteraemia and control cells were analysed and visualised using the scProportionTest package for R (https://github.com/rpolicastro/scProportionTest) (31, 32). Gene set over-representation analysis (GSOA) was performed and visualised using the clusterProfiler and enrichR packages in R (33, 34). Survival analysis was performed using survival data from the GSE65682 (35). Kaplan-Meier plots were generated using the survminer package in R (https://github.com/kassambara/survminer). Module scores were calculated using the AddModuleScore function in Seurat V4 which is calculated using the average expression of each program subtracted by the aggregated expression of control features sets (28). The cytotoxic module score was calculated using cytotoxicity-related genes (*GZMH*, *GZMK*, *GZMA*, *GZMB*, *PRF1*, and *GNLY*). The immunoglobulin score was calculated based on the express of IGHA1-secreted, IGHE-secreted, IGHG1-membrane, IGHG1-secreted, IGHG2-secreted, IGHG3-secreted, IGHG4-secreted, IGHM-membrane, and IGHM-secreted genes. Likewise, the MHC-class II score was calculated based on the expression of *HLA-DMA*, *HLA-DPA1*, *HLA-DQB1*, *HLA-DRA*, and *HLA-DRB1* genes. Single-cell regulatory network Inference and clustering (pySCENIC) was used to infer gene regulatory networks (36). pySCENIC results were visualised using the pheatmap package (v1.0.12).

### 2.6 Statistical analysis

All statistical analyses and visualisations were generated using R (version 4.3.3) and python (version 3.13) software. Significantly differentially expressed genes were determined using an adjusted p-value of < 0.05. Adjusted P-values were calculated using a Bonferroni correction. For across disease comparison, data normality was first assessed using the Shapiro-Wilks test. Subsequent, comparisons were performed using Kruskal-Wallis and Dunn’s tests for non-parametric data, and ANOVA for parametric data. For these comparisons, P-values were corrected for multiple comparisons using a Holm-Bonferroni correction. Levels of significance were denoted as *P-value < 0.05, **P-value < 0.01, and ***P-value < 0.001.

## 3 Results

### 3.1 Multi-omic single-cell sequencing identifies heterogeneous PBMC populations

**Figure 1:**
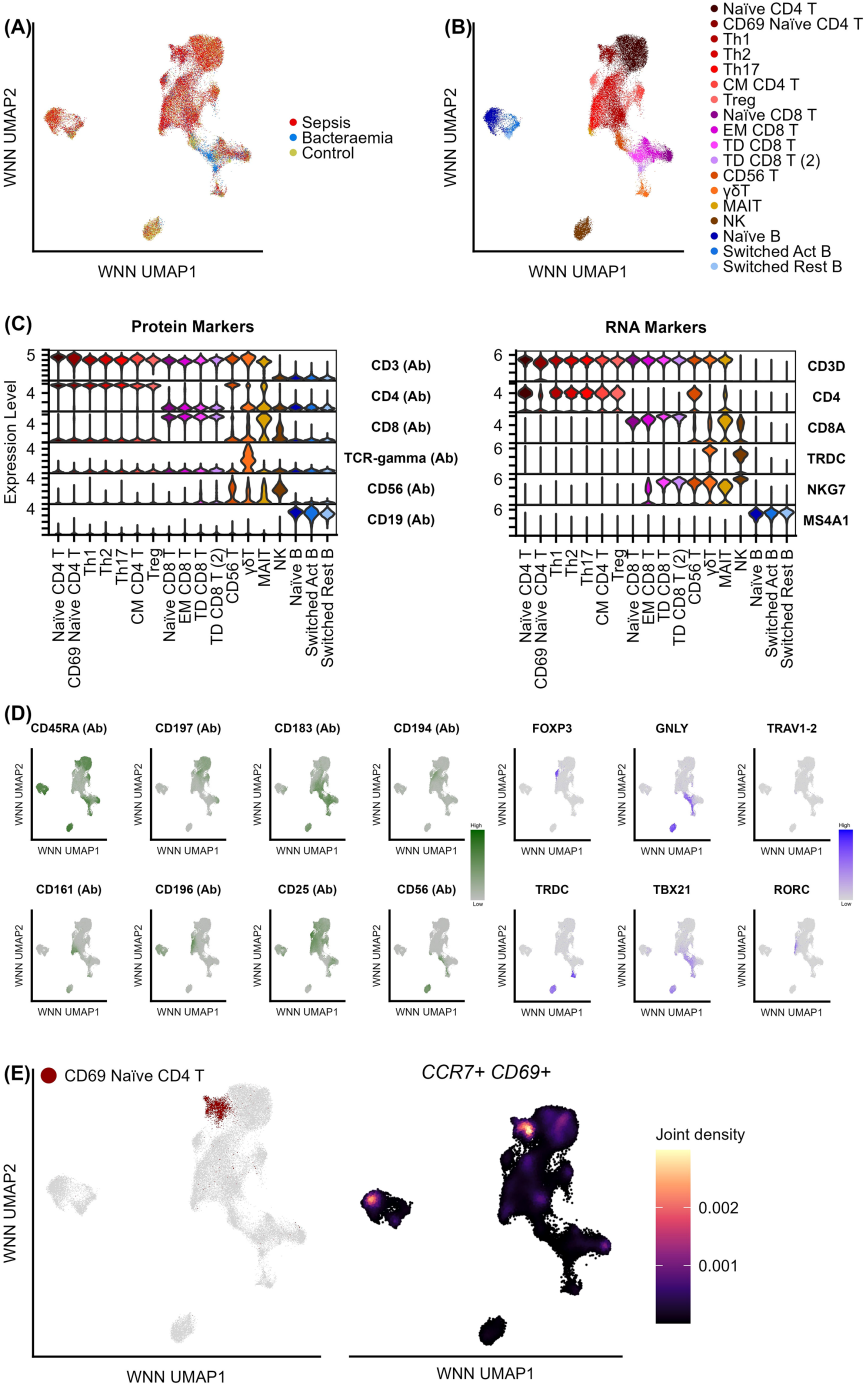
The single-cell atlas of PBMC subsets in sepsis (n = 14), bacteraemia (n = 3), and “healthy” control (n = 3) samples. **(A)** UMAP visualisation of the integration of cells from sepsis (no. of cells = 22091), bacteraemia (no. of cells = 5876), and control (no. of cells = 12269) samples. Each dot on the UMAP plot represents a singular cell. **(B)** UMAP visualisation of the 18 cell types present in the sample cohort. Cell types were identified through unsupervised clustering methods and annotated manually based on established cell markers (**Supplementary Table S4**). **(C)** Violin plots showing the expression of six recognised protein and RNA markers to classify broad cell types. **(D)** UMAP visualisation of the expression of eight recognised protein and six RNA markers used to further annotate specific subtypes of cell populations. **(E)** UMAP visualisation of the CD69+ Naïve CD4+ T cell cluster and density plot of *CCR7* and *CD69* gene co-expression. FDR: false discovery rate.

This analysis incorporated an integrated multi-modal approach to characterise targeted proteomic and transcriptomic expression profiles of immune cells isolated from 14 patients with sepsis, 3 with bacteraemia and 3 healthy controls (**Supplementary Table S1**) using the BD Rhapsody™ Single-Cell Analysis System. Following QC, a total of 40, 236 cells remained for analysis. Cells were characterised into specific PBMC subpopulations based on surface marker and gene expression profiles. The data was integrated to account for batch effects (**Figure 1A**). Using the WNN analysis (28), cells were clustered and assigned a “lowlevel” cell-type annotation, including CD4+ T cells, CD8+ T Cells, γδ T cells, NK cells, and B cells. Monocytes and other myeloid cell types were filtered out due to poor viability post thawing, similarly, remaining myeloid cell clusters were removed from the final analysis. Due to the heterogeneity observed with the major cell types, further clustering at higher resolutions was performed to identify specific populations within each “lowlevel” cell type (**Figure 1B**). A combination of protein markers (Abseq) and gene markers from the literature were used to annotate cell types (**Figures 1C, D**, **Supplementary Table S4**–**6**).

Further sub-clustering revealed 18 distinct cell types, including 14 T cell populations, 1 NK cell population, and 3 B cell populations. Using the multi-modal analysis pipeline, specific subsets of T cell and NK cells were identified through the expression of conventional markers (**Figures 1C, D**, **Supplementary Table S4**) (37–40). Further cell classification was performed on clusters that presented similar expression of conventional markers. This resulted in the identification of two distinct terminally differentiated (TD) CD8+ T cell populations with one population based on the expression of conventional marker EOMES (TD CD8+ T cells) and the other population being defined by NK-like markers KLRC1, KLRC2, and ZNF683 genes (termed TD CD8+ T cells 2) (**Supplementary Table S5**).

Interestingly, two distinct populations of naïve CD4+ T cells were identified based on the expression of naïve T cell markers CD45RA, CCR7 (per Abseq expression) and *CCR7* RNA (**Supplementary Table S4**). Upon further analysis, one population was characterised by the co-expression of naïve T cells markers CD45RA, CCR7 and *CCR7* and the activation marker *CD69*, hereby termed as CD69+ naïve CD4+ T cells (**Figure 1E**). These cells are likely to be recently activated naïve CD4+ T cells.

### 3.2 Sepsis patients have decreased proportions of cytotoxic T cells and increased T helper cell subsets

**Figure 2:**
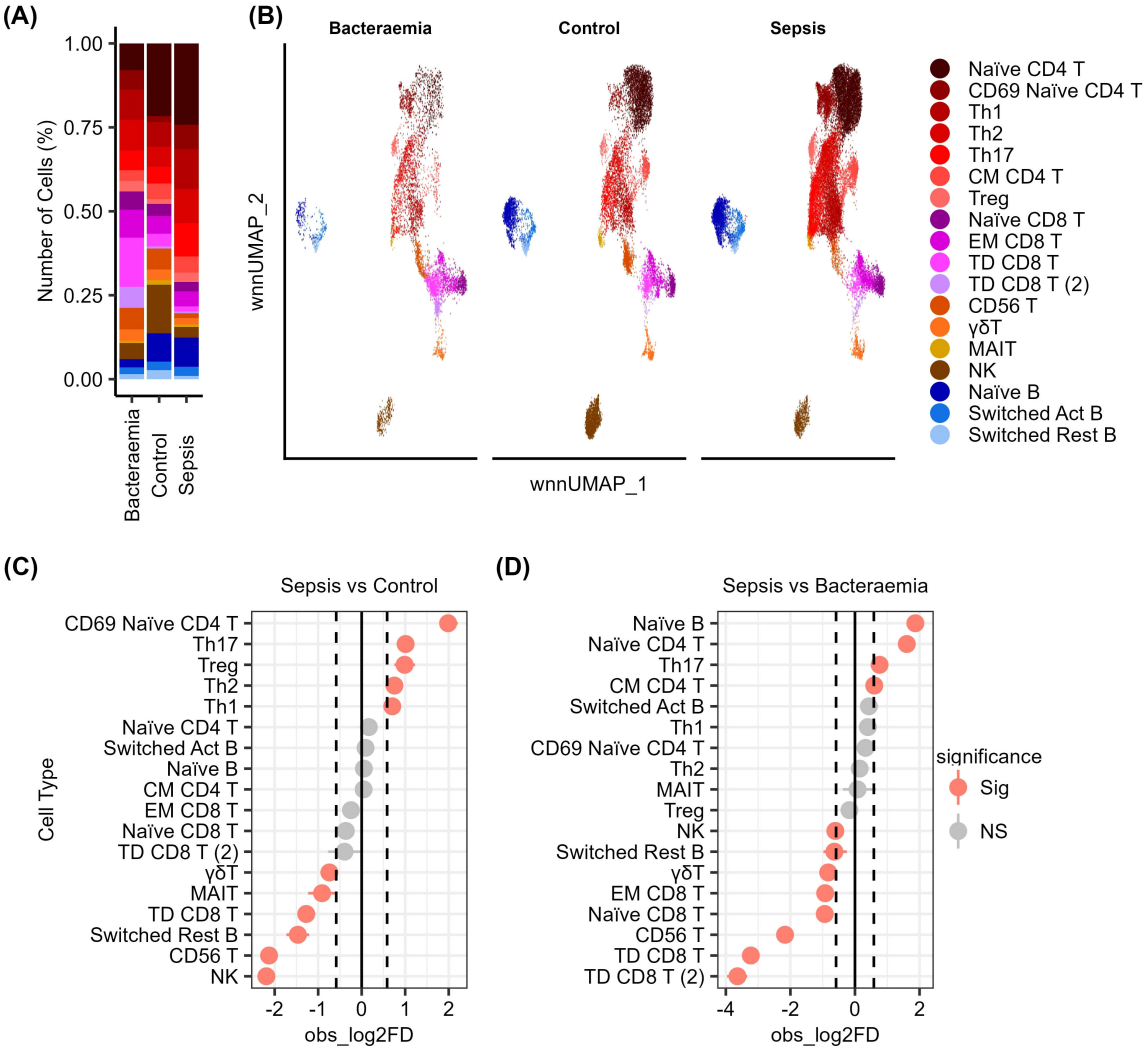
Sepsis induced proportional alterations in several cell subsets. **(A)** The proportion of each cell type in sepsis, bacteraemia, and control samples. **(B)** UMAP visualisation of these proportional differences in each cell type across the sample types. **(C)** Cell type proportional differences between sepsis and control samples. Cell type proportions with an FDR < 0.05 and a Log2 fold change > 0.58 are coloured red and are significantly altered. **(D)** Cell type proportional differences between sepsis and bacteraemia samples.

Control, bacteraemia, and sepsis cell subset abundance is shown in **Figures 2A, B**. Sepsis samples displayed altered proportions of several Th cell subsets compared to bacteraemia and control samples (**Figures 2C, D**). The largest increase in Th proportions in sepsis compared to control samples was in the population of CD69+ naïve CD4+ T cells (1.99log2FC). Other increases observed in sepsis were in Th17 (1.01log2FC), Treg (0.99log2FC), Th2 (0.75log2FC) and Th1 (0.7log2FC) cell subsets compared to control samples. Increases in Th subsets were mirrored in comparisons of sepsis samples to bacteraemia samples with higher levels of naïve CD4+ T cells (1.6log2FC), Th17 cells (0.76log2FC), and central memory (CM) CD4+ T cells (0.59log2FC) (**Figures 2C, D**).

Conversely, PBMCs from sepsis patients displayed decreased proportions of cytotoxic cell subsets compared to bacteraemia and control samples. With decreased proportions of NK cells (-2.19log2FC), CD56+ T cells (-2.13log2FC), terminally differentiated (TD) CD8+ T cells (-1.27log2FC), MAIT cells (-0.91log2FC) and γδ T cells (-0.74log2FC) compared to control samples (**Figure 2C**). Other decreased populations in sepsis included antibody isotype-switched resting B cells (1.47log2FC). Similarly, compared to bacteraemia, sepsis samples maintained lower levels of TD CD8+ T cells (-3.22log2FC), TD CD8+ T cells 2 (-3.63log2FC) CD56+ T cells (-2.17log2FC), EM CD8+ T cells (-0.92log2FC), and γδ T cells (-0.83log2FC) (**Figure 2D**).

### 3.3 Sepsis drives systemic perturbation of immune function

#### 3.3.1 Sepsis *vs.* control populations

**Figure 3:**
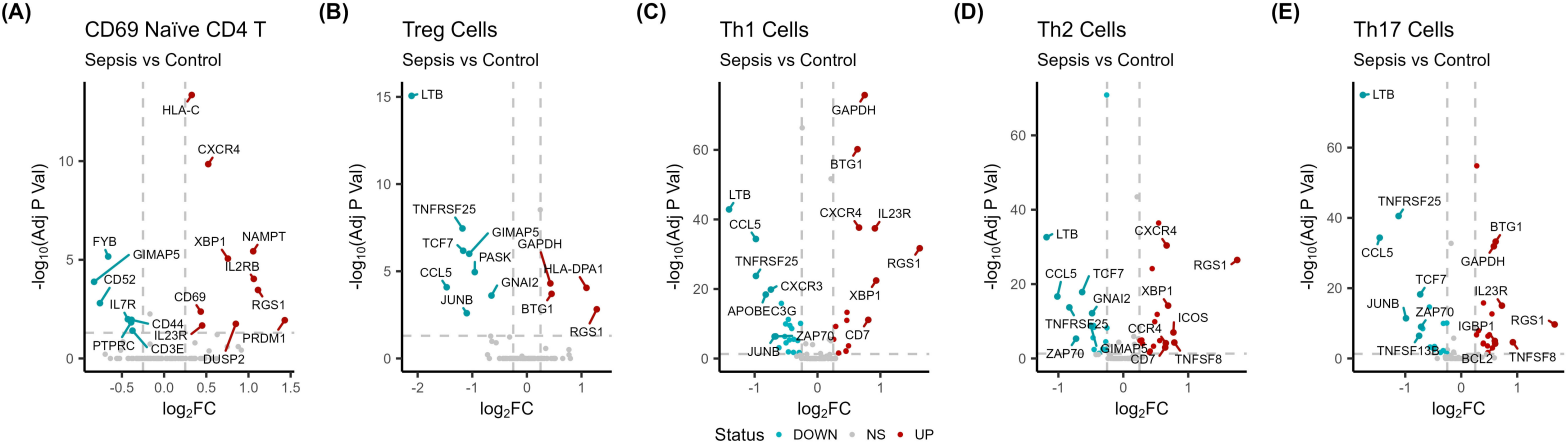
CD4+ T cell dysfunction in sepsis (n =14) patients compared to controls (n = 3). Volcano plots show differentially expressed genes between **(A)** sepsis CD69+ naïve CD4+ T cells and controls, **(B)** sepsis Treg cells and controls, **(C)** sepsis Th1 cells and controls, **(D)** sepsis Th2 cells and controls, and **(E)** sepsis Th17 cells and controls.

**Figure 4:**
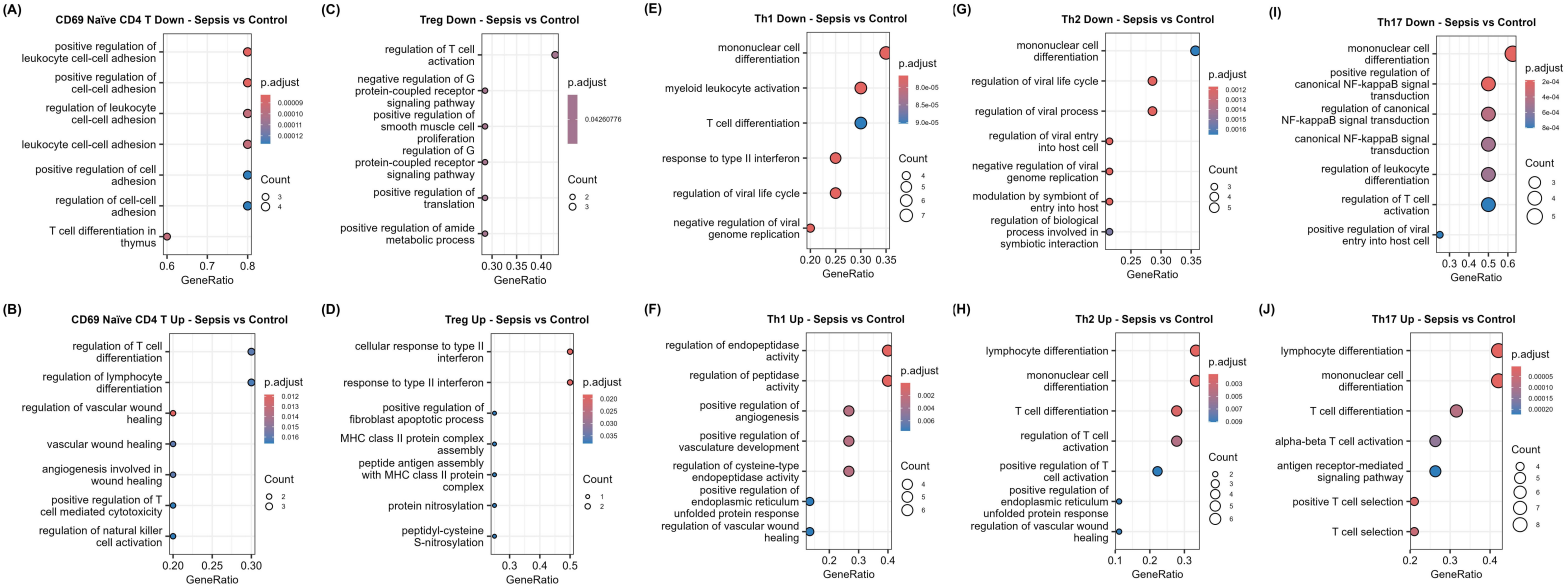
Functional impairment of CD4+ T cells in sepsis patients. GO dotplots of downregulated and upregulated genes in **(A, B)** sepsis CD69+ naïve CD4+ T cells, **(C, D)** Treg cells, **(E, F)** sepsis Th1 cells, **(G, H)** sepsis Th2 cells, and **(I, J)** sepsis Th17 cells compared to control cells.

To further understand what drives the proportional differences in immune cell subsets in sepsis, differential mRNA expression analysis was performed (**Supplementary Table S7**). The largest proportional increase in sepsis patients compared to controls was evident in CD69+ naïve CD4+ T cells. Downregulated genes in CD69+ CD4+ T cells were involved in cell adhesion (*PTPRC*; -0.38log2FC), differentiation (*CD44*; -0.38log2FC), and immunodeficiency (*IL7R*; -0.42log2FC) pathways (**Figure 3A**, **Figure 4A**). This impairment is further highlighted by the downregulation of *GIMAP5*, which is crucial for T cell survival (**Figure 3A**) (44). Conversely, upregulated genes in the CD69+ naïve CD4+ T cell population are involved in T cell differentiation during inflammation and wound healing (*XBP1*; 0.74log2FC) pathways (**Figure 4B**) (41). Several upregulated genes may contribute to T cell exhaustion, including *PRDM1* and *CXCR4* (1.32 and 0.52log2FC, respectively), which have been implicated in a murine model of sepsis (42, 43).

Sepsis Treg cells displayed eight downregulated genes (**Figure 3B**). These downregulated genes were involved in T cell activation (*TCF7* and *JUNB*; -1.1 and -1.0log2FC, respectively) and G protein couple receptor processes (*CCL5*, *PASK*, and *GNAI2*; -1.41, -0.93, and -0.63log2FC, respectively) (**Figures 3B**, **4C**). Interestingly, G protein signalling is important for Treg cell functionality when examined in mouse models (45). In contrast, sepsis Treg cells displayed four upregulated genes (*RGS1*, *HLA-DPA1*, *BTG1*, and *GAPDH*; 1.25, 1.02, 0.44, and 0.43log2FC, respectively) (**Figure 3B**). *RGS1* can impair Treg migration, the class II HLA-DPA1 has been associated with hypoxia in multiple myeloma (46). In addition, *BTG1* is an anti-proliferative gene that is frequently mutated in B cell lymphomas (47), while *GAPDH* participates in glycolysis which displays greater activity during inflammation (48). Together, these upregulated genes are involved in type II interferon and MHC class II-related pathways (**Figure 4D**).

In the Th1 subset, a decrease in the expression of genes involved in cell differentiation (*JUNB* and *ZAP70*; -0.68 and -0.67log2FC, respectively) and type II interferon-related (*CCL5*; -0.98log2FC) pathways was observed (**Figures 3C**, **4E**). These results highlight an impaired functionality in a central T cell population. Conversely the upregulated genes in sepsis Th1 cells showed enhanced endopeptidase activity (*GAPDH*; 0.75log2FC) and angiogenesis related (*XBP1*, *CXCR4*, and *BTG1*; 0.93, 0.66, and 0.64log2FC, respectively) pathways (**Figure 4F**). Together, this data indicates Th subset activation, differentiation and functionality is undermined by a diverse and complex mechanism of functional impairment during sepsis.

Downregulated genes in sepsis Th2 cells were significantly overrepresented in processes related to viral responses (including *TNFRSF25* and *GIMAP5*; -0.79 and -0.49log2FC, respectively) and differentiation (*ZAP70*; -0.66log2FC) (**Figures 3D**, **4G**). Similarly, sepsis Th2 cells displayed upregulation of genes involved in T cell activation (*XBP1*; 0.66log2FC) and differentiation compared to control samples (**Figures 3D**, **4H**). Additionally, *RGS1* (1.67log2FC) was the most upregulated gene, indicating a migratory impairment to effector Th cells.

Differential expression in Th17 cells in sepsis compared to controls identified downregulation in genes associated with T cell activation, differentiation (*JUNB*; -0.90log2FC), and inflammatory pathways, including NF-κB signalling. These included several immune effector response genes, such as *IFITM2*, *IFITM3*, and *APOBEC3G* (-0.51, -0.49, and -0.46log2FC, respectively) (**Figures 3E**, **4I**). Such downregulation suggests the expansion of functionally impaired Th17 cells in sepsis. Conversely, upregulated genes were involved in differentiation (*TNFSF8* and *BCL2*; -0.83 and 0.54log2FC, respectively), activation, and T cell selection (**Figures 3E**, **4J**).

#### 3.3.2 Sepsis *vs.* bacteraemia populations

**Figure 5:**
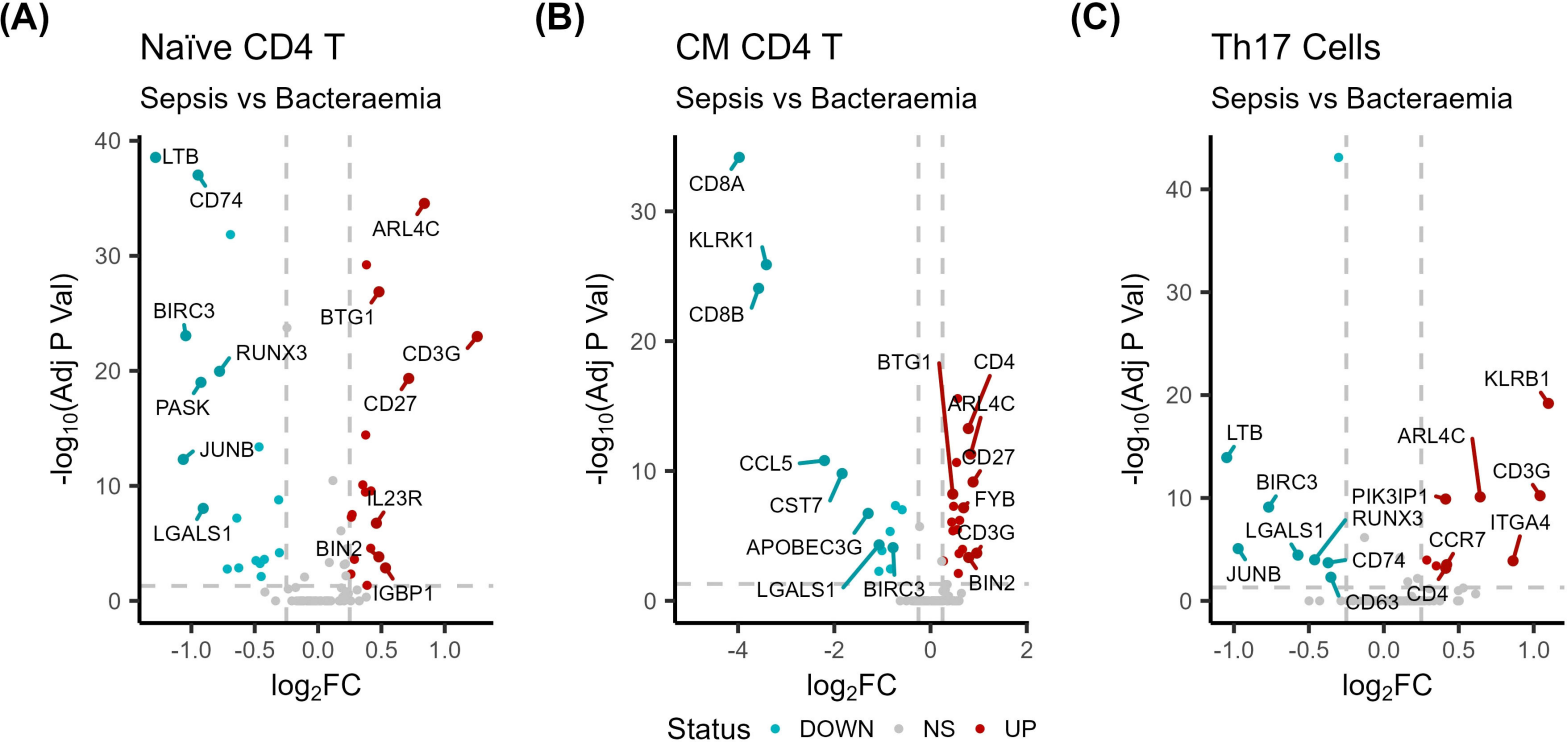
Sepsis helper T subsets are functionally impaired compared to bacteraemia cells. Volcano plots of differentially expressed genes in **(A)** Naïve CD4+ T Cells, **(B)** CM CD4+ T cells **(C)** Th17 cells from sepsis (n = 14) patients compared to controls (n =3).

**Figure 6:**
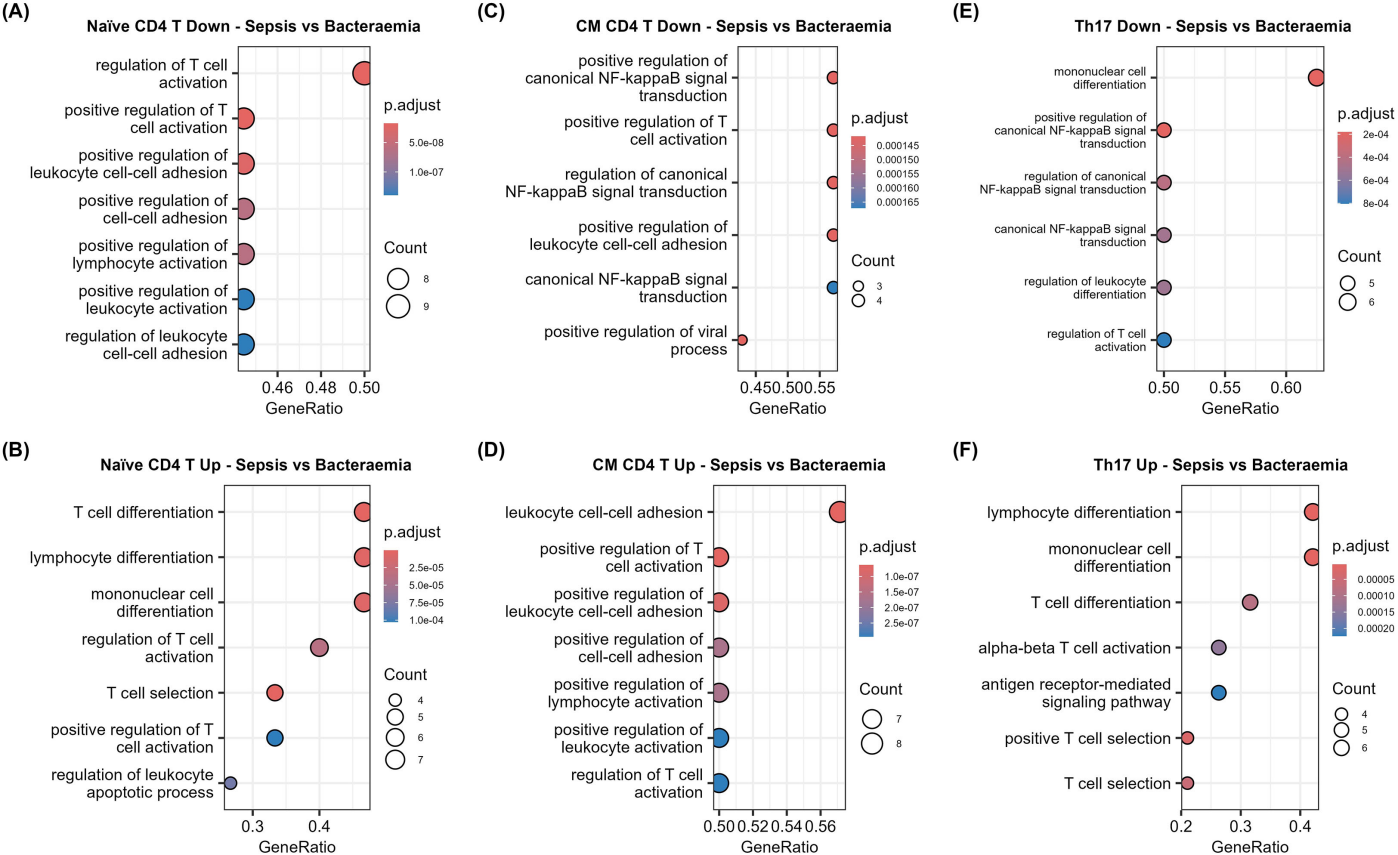
Impaired activation and functionality in helper T cell subsets in sepsis (n = 14) compared to bacteraemia (n =3). Dotplots displaying significantly altered GO BP that are **(A)** downregulated and **(B)** upregulated in sepsis naïve CD4+ T cells, **(C)** downregulated and **(D)** upregulated in sepsis CM CD4+ T cells, and **(E)** downregulated and **(F)** upregulated in sepsis Th17 cells compared to bacteraemia samples.

Sepsis samples were compared to bacteraemia samples (**Supplementary Table S8**). Naïve CD4+ T cells represented one of the largest proportional increases in sepsis compared to bacteraemia samples (1.6log2FC). The downregulated genes were overrepresented in T cell activation (*JUNB*, *LGALS1*, and *RUNX3*; -0.98, -0.87, -0.76log2FC, respectively) and cell adhesion processes (**Figures 5A**, **6A**). Conversely, upregulated genes in these cells are known to participate in differentiation (*CD27* and *IL23R*; 0.70 and 0.45log2FC, respectively) and apoptosis (**Figures 5A**, **6B**).

CM CD4+ T cell proportions were increased in sepsis compared to bacteraemia. Transcriptional analysis highlighted the downregulation of NF-κB signalling (*LGALS1* and *BIRC3*; -1.07 and -0.78log2FC, respectively), activation (*CCL5*; -2.20log2FC)., and cell adhesion processes (**Figures 5B**, **6C**, **Supplementary Table S8**) On the other hand, immune regulatory genes *BTG1* (0.46log2FC), *PIK3IP1* (0.44log2FC), T cell activation markers *CD27* (0.86log2FC), *FYB* (0.67log2FC), and *TRAT1* (0.56log2FC) were upregulated in sepsis CM CD4+ T cells (**Figure 5B**, **6D**, **Supplementary Table S8**) (49–51). Interestingly, CM CD4+ T cells also displayed upregulation of cell adhesion genes (*CD27* and *ARL4C*; 0.88 and 0.83, respectively). Together, these changes demonstrate the complexity of sepsis-induced impairments.

Th17 cell alterations have been highlighted in sepsis and are associated with poor prognosis (52). The elevated proportion of sepsis Th17 cells in this dataset compared to bacteraemia samples displayed downregulation of genes involved in cell differentiation (*RUNX3*; -0.45log2FC), NF-κB signalling (*LTB*, *BIRC3*, *LGALS1*, and *CD74*; -1.0, -0.75, -0.56, and -0.36log2FC, respectively) and activation pathways (**Figures 5C**, **6E**). Upregulated genes identified in sepsis Th17 cell compared to bacteraemia cells were involved in T cell differentiation (*ITGA4* and *CCR7*; 0.80 and 0.41log2FC, respectively), activation, and antigen receptor signalling (*CD3G*; 0.98log2FC) pathways (**Figures 5C**, **6F**).

### 3.4 Sepsis induces widespread impairments to cytotoxic activity

Cytotoxic cell populations were significantly reduced in sepsis samples compared to control and bacteraemia samples. Reductions in NK cells (-2.19 & -0.61log2FC), TD CD8+ T cells (-1.27 & -3.23log2FC), γδ T cells (-0.74 & -0.83log2FC), and CD56+ T cells (-2.13 & -2.17log2FC) were evident in sepsis compared to both control and bacteraemia samples (**Figure 2C**).

**Figure 7:**
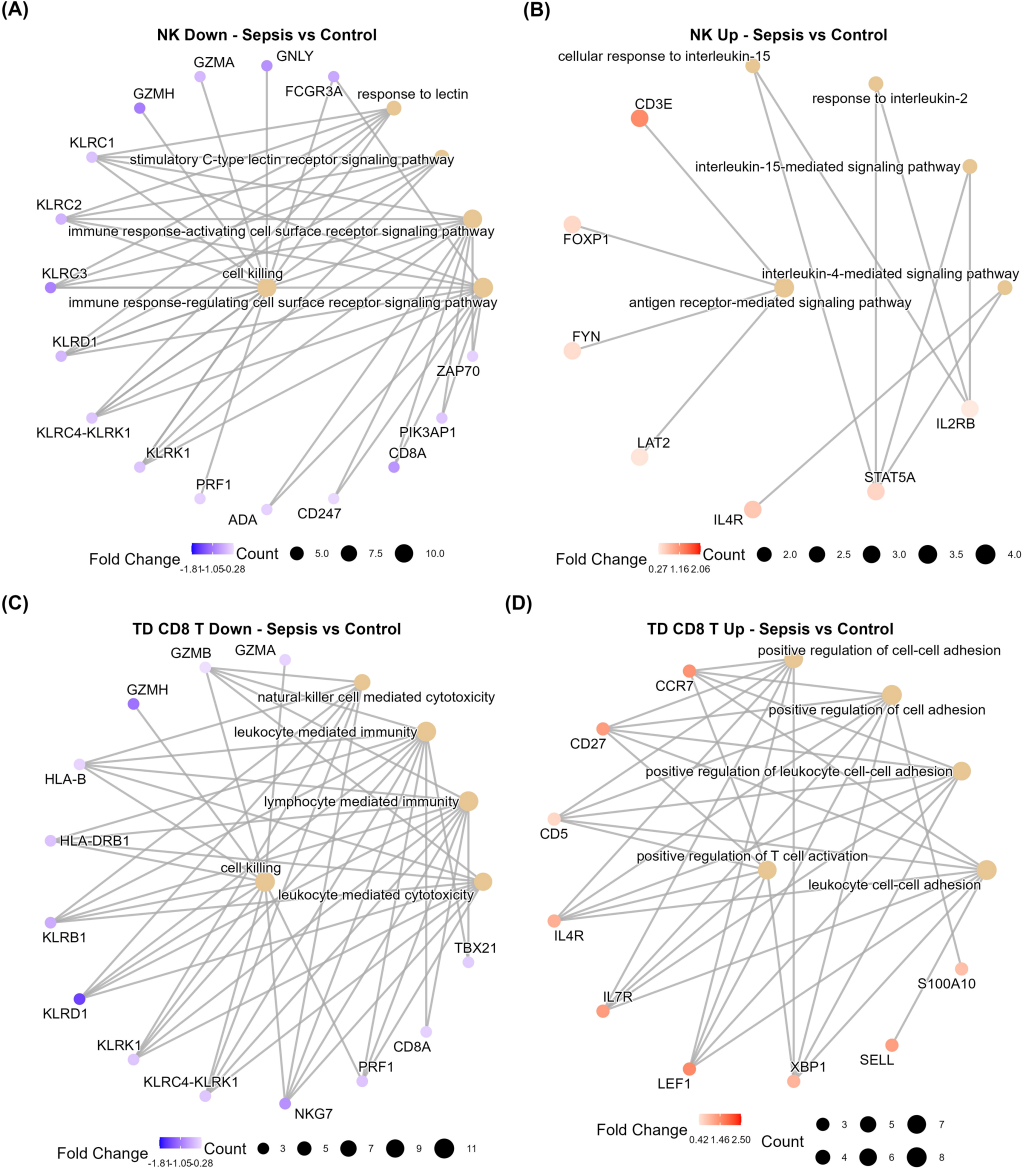
Diminished cytotoxic capacity in sepsis compared to control samples. CNetplots displaying genes and their associated differentially regulated GO BP in **(A)** downregulated in sepsis NK cells compared to control NK cells, **(B)** upregulated in sepsis NK cells compared to control NK cells, **(C)** downregulated in sepsis TD CD8+ T cells compared to control TD CD8+ T cells and **(D)** upregulated in sepsis TD CD8+ T cells compared to control TD CD8+ T cells.

Differential expression analysis of sepsis NK cells highlighted further immunosuppression through the downregulation of critical genes involved in target cell killing such as granzyme A (GZMA), lectin response, as well as antigen processing and presentation pathways compared to controls (**Figure 7A**). Despite impaired cytotoxicity, sepsis NK cells maintained upregulation of genes involved in cytokine-mediated, and antigen receptor-mediated signalling compared to controls (**Figure 7B**). These findings suggest that although NK cell cytotoxic capacity is severely impaired, their ability to respond through cytokine secretion may remain functional during sepsis.

Compared to bacteraemic NK cells, sepsis NK cells maintained a level of cytotoxicity gene expression, including *GZMA* and *PRF1* (1.03log2FC & 0.53log2FC) (**Supplementary Table S8**). Furthermore, sepsis NK cells displayed upregulation of migration, chemotaxis, and immune response processes, indicating effective NK cell function (**Supplementary Figure S1B**). However, downregulated genes in sepsis NK cells similarly included cytotoxicity-related genes (*GZMH*, *GNLY* & *GZMB*, -1.42log2FC, -1.29log2FC & -0.48log2FC, respectively) (**Supplementary Figure S1**). Downregulated genes were significantly overrepresented in cell activation, adhesion, antigen processing and presentation, including downregulation of *HLA-DRB1* and *HLA-DRA* (-1.81log2FC & -1.00log2FC, respectively) (**Supplementary Figure S1**). In contrast, *HLA-DR* expressing NK cells are a functionally distinct population of NK cells that effectively produce pro-inflammatory cytokines, degranulate and proliferate in response to infections (53, 54). These results capture the multifaceted immunosuppressive effects sepsis induces in NK cells.

TD CD8+ T cells were significantly decreased in sepsis compared to both control and bacteraemia samples. Compared to controls, sepsis TD CD8+ T cells displayed downregulated genes that were overrepresented in cell killing and antigen presentation (**Figure 7C**). Conversely, upregulated genes were involved in cell adhesion and T cell activation (**Figure 7D**). This impairment of cytotoxicity is mirrored in sepsis TD CD8+ T cells when compared to bacteraemia samples (**Supplementary Figure S1**). Similarly, when compared to bacteraemia cells, sepsis TD CD8+ T cells displayed upregulation of genes involved in cell adhesion and regulation of T cell activation, while maintaining a similar decrease in cell killing, and cytotoxicity processes (**Supplementary Figure S1**).

**Figure 8:**
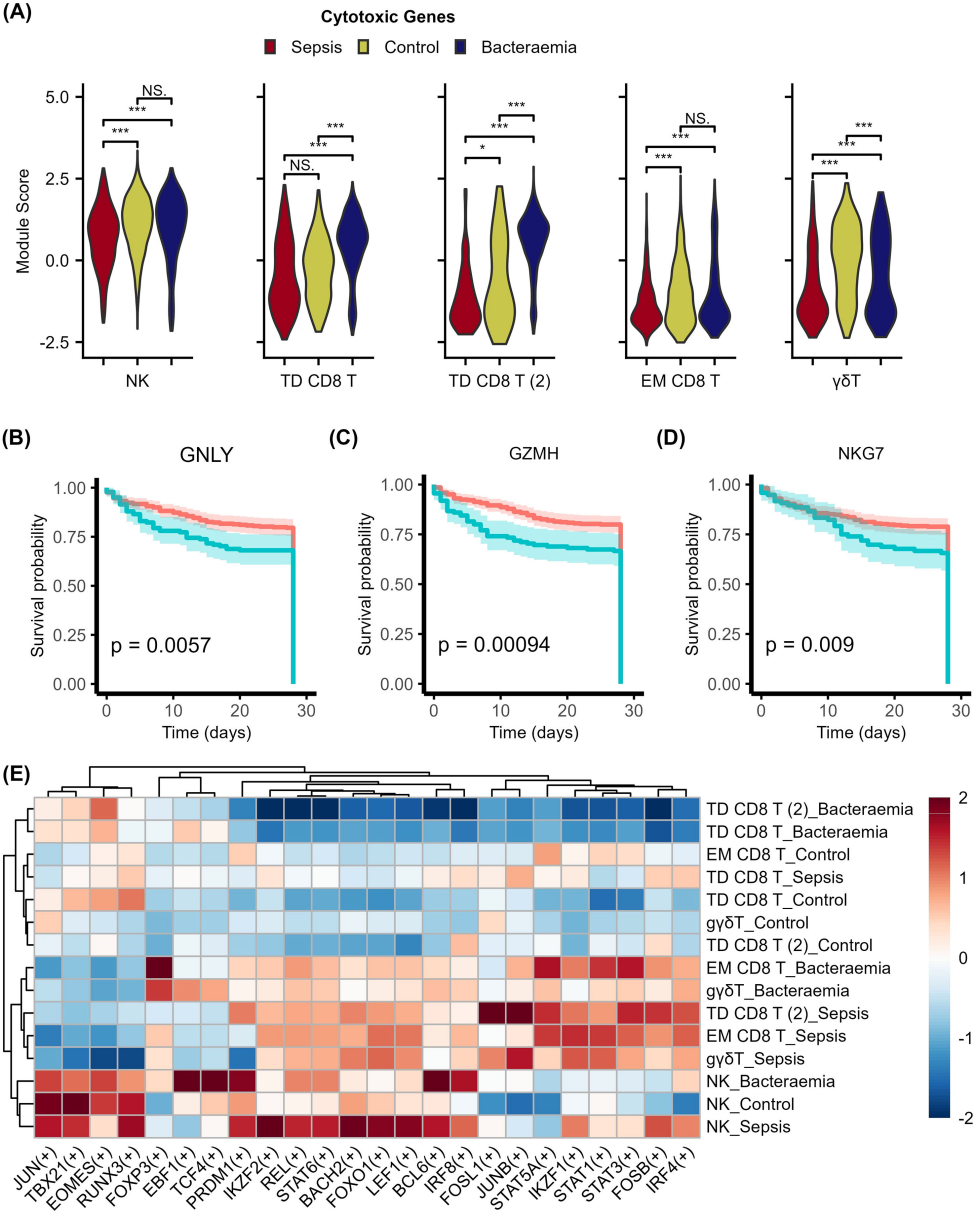
Impaired cytotoxic capability in sepsis is associated with worse outcomes. **(A)** Violin plot displaying a cytotoxic module score calculated based on the expression of several cytotoxicity-related genes (*GZMA*, *GZMB*, *GZMH*, *GZMK*, *GNLY*, & *PRF1*) in the cytotoxic cell populations using the AddModuleScore function in Seurat V4. Cytotoxicity scores were compared between sepsis, bacteraemia, and controls for NK cells, TD CD8+ T cells, EM CD8 T and γδT cells. **(B-D)** Kaplan-Meier plots highlighting the association of decreased expression of cytotoxic genes and poor survival outcomes in sepsis. **(E)** Scaled heatmap of gene-regulatory activity in cytotoxic cell subsets. NS: not significant, *P-value < 0.05, **P-value < 0.01, ***P-value < 0.001.

The proportion of γδ T cells decreased in sepsis compared to both control and bacteraemia samples (**Figures 2C, D**), mirroring previous studies (55, 56). In addition, the downregulation of cytotoxic-related genes in sepsis γδ T cells compared to control and bacteraemia cells, illustrate the loss of cell-killing capabilities of this key cell type during sepsis (**Supplementary Tables S7, 8**). In addition, these results are strengthened by a significantly lower cytotoxic module score (calculated using the AddModuleScore function in Seurat V4 using the cytotoxic-related genes *GZMH*, *GZMK*, *GZMA*, *GZMB*, *PRF1*, and *GNLY*) in all sepsis cytotoxic cell subsets compared to bacteraemia and controls (**Figure 8A**). This is further reinforced by the reduced survival in sepsis patients with downregulated cytotoxic-related genes (**Figures 8B–D**).

To further investigate this widespread inhibition of cytotoxic activity, a transcription factor (TF) and regulon analysis was performed. Focusing on these cytotoxic cell subsets, **Figure 8E** highlights differences in regulon activity between the control, bacteraemia, and sepsis cytotoxic subsets. Clustering of these disease-specific cell types reveals separation of bacteraemia-related cell types from sepsis and control cell types. These regulons include important functionality-related TFs including *FOXO1* and *BACH2*, and exhaustion-related TF *IKZF1*. Regulons typically involved in cytotoxicity include *TBX21*, *EOMES*, *RUNX3*, and *JUN*, as identified through the pySCENIC analysis. Subsequent analyses of these regulons area under the curve (AUC) highlighted significantly altered regulon transcriptional activity in sepsis cytotoxic cell populations compared bacteraemia and control populations (**Supplementary Figure S2**).

### 3.5 Sepsis impacts B cell antigen presentation, antigen processing, and immunoglobulin production

**Figure 9:**
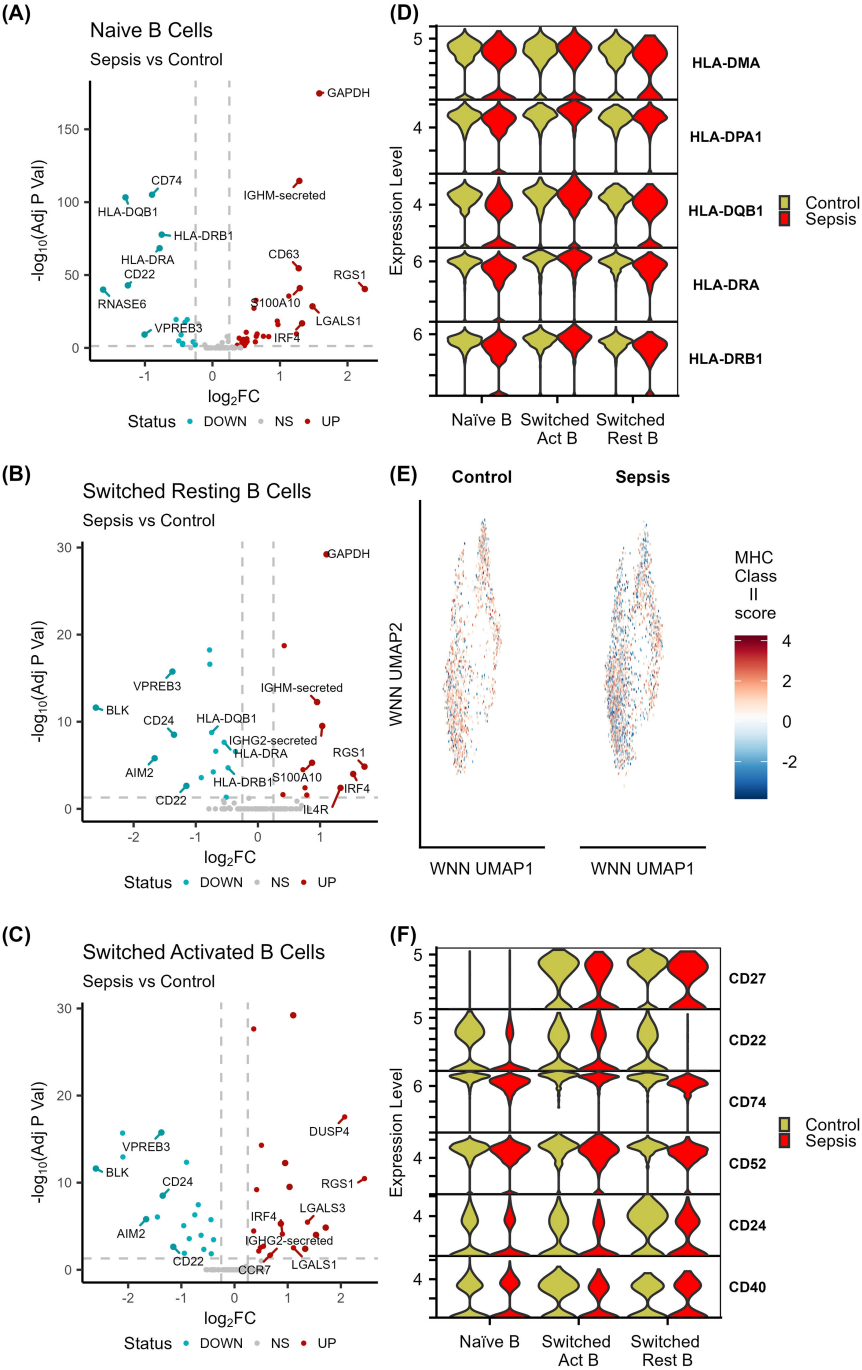
Impaired B cell responses in sepsis. **(A-C)** Volcano plots depicting differentially expressed genes between sepsis and control naïve B cells, switched resting B cells, and switched activating B cells. **(D)** Comparison of HLA-class II gene expression between sepsis B cells and control B cells. **(E)** UMAP visualisation of HLA-class II gene module score in sepsis and control B cells. **(F)** Comparison of B cell activation and development genes in sepsis and control B cells.

Our understanding of B cell alterations during the disease course of sepsis is limited. Sepsis samples exhibited decreases in switched resting B cells compared to both control and bacteraemia samples. They also displayed increased proportions of naïve B cells compared to bacteraemia samples (**Figures 2C, D**). When comparing sepsis B cells to control cells, 45 genes were differentially expressed in naïve B cells, 27 genes in switched resting B cells and 28 genes in switched activated B cells (**Figures 9A–C**). In sepsis *RGS1*, along with *IRF4* and *GAPDH*, were highly upregulated across all B cell populations compared to control cells. This upregulation of *RGS1* was complemented by the downregulation of *GNAI2* in switched resting and switched activated B cells. These two genes have previously been implicated in impaired B cell trafficking and motility within peripheral lymph nodes (57). Similar to T cells and NK cells in sepsis, both naïve and switched resting B cell populations display significant decreases in the expression of several MHC-class II genes, including *HLA-DRA*, *HLA-DRB1*, *HLA-DMA* and *HLA-DPA1* (**Figure 9D**). Furthermore, several immunoglobulin genes including membrane and secreted forms of *IGHM*, *IGHG1* and *IGHG2* were also reduced in sepsis patients. Calculation of an immunoglobulin module score in the B cell populations further confirmed these alterations, particularly between naïve and switched activated B cells (**Figure 9E**, **Supplementary Figure S3**). Interestingly, marked alterations in B cell activation (*CD27*, *CD22*, and *CD74*) and development markers (*CD52*, *CD24*, *CD40*) were observed across B cell populations. In particular, significant decreases were evident in naïve B cells (*CD22*, *CD74*, and *CD52*), switched resting B cells (*CD24*, *CD22*, *CD74*, *CD52*, and *CD27*), and switched activated B cells (*CD40*, *CD27*, and *CD52*) (**Figure 9F**). Together, these data illustrate the downregulation of central B cells activation, differentiation, and immunoglobulin production in sepsis compared to control cells (**Figures 9E, F**, **Supplementary Figure 5**).

## 4 Discussion

In the current study, we utilise a custom-targeted panel of 46 Abseq protein and 424 mRNA markers to characterise PBMC-specific cell populations and explore differences in PBMCs between sepsis, bacteraemia, and healthy controls. By combining the concurrent measurement of mRNA and protein markers on immune cells and using the weighted multi-modal approach (28), we have provided a comprehensive analysis of specific cell subtypes. This would otherwise have been difficult to categorize using a single mRNA or protein approach alone. Using this integrated approach, we have highlighted the alterations of T and B cell populations across each disease state compared with healthy controls. Across the three conditions, 18 different PBMC populations were identified.

This study identified a distinct population of CD69+ naïve CD4+ T cells. In healthy individuals, these CD69 expressing naïve CD4+ T cells represent approximately 2-11% of total naïve CD4+ T cells (58). The identification of this rare subset, highlights the utility of our multi-modal approach. Studies examining this population show increased proportions of this cell type in several autoimmune pathologies (59). While the expression of *CD69* was upregulated during sepsis, the increased CD69+ naïve CD4+ T cell population has not been reported previously in this condition (60). Although these cells are primed for a rapid and strong immune response, the sepsis population identified in our study displayed an impaired phenotype due to the expression of *CCR7* (58). Our data raises the prospect that CD69+ naïve CD4 T cells may be a useful biomarker in the diagnosis of sepsis. While other reports shows that CD69+ T helper cells are increased in mouse models of sepsis (60, 61), this is the first paper that shows this phenomenon in sepsis patients. CD69 is a type II C-lectin membrane bound receptor and is an early marker of T cell activation (62). Its expression is switched on 30 minutes following TCR engagement and is induced by several TFs including NF-κB, erythroblast transformation-specific related gene-1 (ERG-1) and activator protein-1 (AP- 1) (62). Signalling downstream of CD69 results in the activation of STAT5 and blockade of S1P1 signalling to promote Treg development and to limit Th1 and Th17 responses (62). CD69 is expressed on several T cell populations including CD8+, CD4+, Treg and γδ T cells and is required for the formation of resting T-helper memory cells (62). While the expression of CD69 is regarded as a marker of tissue retention, its role in circulating T cells such as CD4+ T cells is poorly defined and thus requires further investigation. In mice it was shown that these cells exhibit more rapid and greater production of IL-2 and TNF-α compared to CD4+ T cells lacking CD69 (58). If this is the case in the human system also, it may indicate that CD69 drives a more inflammatory phenotype and a possible pathological response of this cell population in sepsis. Further investigation is warranted to assess the functional impact of this subset of naïve CD4+ T cells in the development of sepsis and as well as post sepsis recovery.

Similarly, when comparing sepsis cells to both bacteraemia and control cohorts, it is interesting to note the decrease in the proportion of several cytotoxic T and NK cell populations. This evident decrease in cytotoxic T cell population and function has been reported previously, emphasising an immunoparalysis state experienced during the sepsis disease course (63–65).

It is unclear at present what factors govern why some patients who with bacteriaemia go on to develop sepsis and why others do not. This may be due to the infection type, the burden of microbes present or host specific factors. Importantly, sepsis is a highly variable clinical syndrome caused by a dysregulated host response to infection with variable presentations and severity. In this study, the participant cohorts were defined using established clinical criteria (Sepsis-3 definition of a SOFA score ≥2), whereas bacteraemia without sepsis has positive blood cultures, but did not meet the sepsis criteria (had SOFA scores <2 at time of sampling) (1). These represent distinct clinical populations, however, it should be recognised that this may not fully capture the complexity of host responses to infection. Nonetheless, our cohort did not include patients with blood culture positive sepsis, and this should be considered when interpreting these findings.

In the past apoptosis induced by excessive cytokine production and PD-1 upregulation was thought to be the dominant form of cell death in sepsis, however it is now appreciated that release of host DAMPs such as ATP leading to pyroptosis is an important driver of pathology (10, 66). Recent work has shown that LPS can combine with haem from red blood cells to drive a novel form of cell death known as PANoptosis in macrophages (67). Whether this form of cell death also occurs in cytotoxic lymphoid cells has yet to be addressed. These differences in the comparison of bacteraemia to sepsis have not been fully described. Whatever factors dictate the threshold for cytotoxic cell death that is surpassed in sepsis are not present in bacteraemia patients.

As previously detailed, several populations of cells, including Th17 and Th1 cells, were significantly decreased in the sepsis cohort. These proportional dynamics have been observed in other studies (25, 52). However, in our study, the differential expression analysis suggests that although sepsis samples maintained increased proportions of effector CD4+ T cell populations, these populations displayed a complex impaired expression profile compared to control populations. A common feature of the Th subset in sepsis is altered antigen processing and presentation capabilities (68), and is further highlighted in this study. It is possible that MHC class II expression by Th cells in our dataset reflects T cell activation rather than antigen presentation, with the latter being more typically associated with B cells. As a previous study suggests the frequency of HLA expressing T cells are related to T cell activation in those with hyperinflammatory disorders (69). Nonetheless, the necessity of antigen-specific engagement for full T cell activation in sepsis should be considered, as TCR engagement alone does not fully activate the T cells (70). The loss of HLA genes reported here may prevent T cells from antigen-dependent activation, thus impairing an effective immune response. However, further investigation is warranted to confirm the underlying cause of this loss of HLA expression.

As expected, the PBMCs from sepsis displayed proportional decreases in several important effector T cell populations (**Figures 2A, B**), compared to both bacteraemia and healthy controls. Of the remaining cytotoxic immune cells present in sepsis patients, these cells displayed a clear reduction of in genes required to execute cytotoxic functions. Effective cytotoxic action is central to the clearance of intracellular pathogens, including various bacteria, viruses, and fungal pathogens (71, 72), and their impaired function in sepsis clearly explains the enhanced susceptibility of sepsis survivors to subsequent infection and re-hospitalisation.

In this study, our data suggest that the downregulation of key differentiation-related genes, such as *KLRB1* and *HLA-DRB1*, may contribute to this decrease in CD8+ T cell function compared to both control and bacteraemia cohorts. Furthermore, the widespread downregulation of specific cytotoxic and cytolytic genes, including *GNLY*, *GZMH* and *NKG7*, across CD8+ T cells, NK cells, CD56+ T cells, MAIT cells and γδ T cells reported here was found to negatively impact survival outcomes in sepsis. The subsequent analyses highlighted the potential role for altered TF activity in contributing to this impairment. The clustering of sepsis and control cell types together is interesting as it demonstrates a differentially regulated response to infection compared to that of bacteraemia. Furthermore, the TF analysis highlighted increased regulon activity of important functionality-related TFs (*FOXO1* and *BACH2*). Particularly in NK cells, these TFs negatively regulate NK cell maturation and function, which may contribute to this sepsis-related impairment (73, 74). Likewise, when focusing on TFs related to the regulation of cytotoxicity genes (*TBX21*, *EOMES*, *JUN*, and *RUNX3*), alterations are evident within the sepsis cells. While sepsis NK cells maintained *TBX21* activity, *EOMES* activity was significantly downregulated in sepsis. This may explain the persistence of partial cytotoxic capability evident in sepsis NK cells as both are required for efficient NK cell function (75, 76).

Similar observations were found in CD8+ T cell subsets, with decreased activity in cytotoxic-related TFs in sepsis. The decrease in *JUN* and *TBX21* in the sepsis population of TD CD8+ T cells may contribute to impaired cytotoxic capability through the development of a dysfunctional state. The *JUN* TF is part of the AP-1 TF complex, which typically dimerises with the nuclear factor of activated T cells (NFAT) to control transcription of important functional and cytotoxic genes. However, loss of AP-1 can result in NFAT alone promoting an exhausted CD8+ T cell phenotype with diminished cytotoxicity (77, 78). Likewise, a decrease of *TBX21*, evident in sepsis TD CD8+ T cells, can cause impaired cytotoxicity (79, 80). This holds true for the sepsis population of TD CD8+ T cells 2 which display a decrease in all four TFs compared to bacteraemia cells (80), likely compounding this impairment. The combined decrease in cytotoxic cells and the concurrent diminished cytotoxic function of the remaining cytotoxic cells clearly contributes to the recurrent infections associated with sepsis, as the body fails to clear infection effectively (81). Together, this decreased cytotoxic signature may have prognostic implications for those with sepsis, as diminished cytotoxic capacity has been linked to adverse outcomes in independent sepsis cohorts (82, 83).

This study also characterised naïve and memory B cell populations in sepsis compared to control and bacteraemia and highlighted increased levels of naïve cells and decreases in switched resting cells in sepsis. These alterations in B cell proportions are likely driven by significant cell death mediated by apoptosis and pyroptosis as described above for the cytotoxic cells. Our findings are supported by other work, which has shown that B cell death is associated with sepsis and reduced survival (5, 17). In addition to B cell death, naïve and switched B cells display downregulation of MHC-class II genes, such as *HLA-DQB1*, *HLA-DRA*, and *HLA-DRB1*, which serve as both a means of antigen processing and presentation, and B cell activation, differentiation and proliferation (84). These data are also supported by other studies that have shown decreased expression of *HLA-DR* in B cells during septic shock (85, 86). Interestingly, *HLA-DR* is downregulated in trauma patients that experienced subsequent infection compared to those who did not (85). The impairment mechanism observed in B cells during sepsis might be further explained by the loss of important activation markers and proliferation genes such as *CD22*, *CD27* and *CD74* (87, 88). Therefore, this study advances our understanding of B cells during sepsis pathobiology by providing further evidence of a more widespread loss of MHC-class II expression and impaired antigen processing and presentation capabilities in naïve and switched resting B cells.

The B cell impairment reported here would explain the decreased immunoglobulin levels observed in patients with sepsis. Previous literature indicates higher sepsis-related mortality in people with diminished immunoglobulin levels, specifically reduced levels of IgA, IgG, and IgM (89, 90). However, such decreases were highly variable among different patients and is reflected in our study where we observed widespread downregulation of immunoglobulin genes in sepsis B cells (**Supplementary Figure S5**). Alterations in immunoglobulin expression were observed only in naïve B cells and switched activated B cells (**Supplementary Figure S3**). The lack of differences observed in switched resting B cells suggests that the issue of immunoglobin expression may result from impaired activation.

The limitations of this study include initial cell viability, which led to reduced cell numbers. The sepsis and bacteraemia samples were frozen for approximately 2–3 years and thawed prior to analysis. During this process, most myeloid cells were lost due to poor viability post recovery. Consequently, no characterisation of myeloid cells was performed, and such viability issues may have resulted in the underrepresentation of certain cell populations. Previous literature has demonstrated a disproportionate reduction in myeloid cell population and alteration of their transcriptome following cryopreservation which may explain the low recovery of monocytes in this study (91). Nonetheless, modification of the pipeline allowed for the examination of valuable retrospective samples, demonstrating its utility for immunological investigation.

As is the case for many single-cell sequencing studies, batch effects can be a concern. To mitigate this, we applied a conservative approach using Seurat’s RPCA integration to prevent over-correction and preserve condition-specific biology, however such methods can still over-correct. It is important to note that the bacteraemia without sepsis samples displayed distinct gene expression profiles compared with ‘healthy’ donors, as evidenced by the increased cytotoxic score in bacteraemia samples. However, ‘healthy’ donors were not tested for transient bacteraemia. While the exclusion criteria minimised the risk of infection in the controls, infection cannot be fully excluded. Also, the use of a targeted panel limits the analysis to the immune response specifically and does not cover other possible relevant genes, such as those involved in metabolism.

Finally, this study is limited by a relatively small cohort size, which is consistent with other recent single-cell studies of sepsis. While this restricts the studies statistical power, it reflects the common constraints of single-cell sequencing studies, which have been limited to pilot-scale studies dues to cost, technical complexity, and challenges in sample accrual (22). Furthermore, while the expansion of CD69^+^ naïve CD4^+^ T cells in sepsis is interesting, it may reflect cohort-specific factors and requires validation in larger, independent cohorts. Comorbidities were present in our patient population, as is typical of sepsis cohorts. and although we reported detailed demographic and severity indices (**Supplementary Table S1**), our study was not powered to stratify by comorbid condition. Sepsis is a highly heterogeneous condition, influenced by patient demographics, comorbidities, and clinical context. While our cohort captures some of this variability, it does not encompass the full spectrum of patients at risk of or affected by sepsis. As such, our findings should be regarded as hypothesis-generating and require validation in larger, more diverse cohorts. Larger multicentre studies will be critical to further define the impact of comorbidities and other host factors on immune responses in sepsis.

Through the integration of targeted concurrent proteomic and transcriptomic data, this study identified alterations in cell function within Th and cytotoxic compartments. Thus, the data presented here extends our understanding of sepsis in several unique ways as it provides a comprehensive global overview of the immune system defects in sepsis. Consequently, we have reported a population of *CD69* expressing naïve CD4+ T cells for the first time in sepsis, which advances our understanding of sepsis pathophysiology. We show increased proportions of this CD69+ naïve CD4+ T cell population, increased effector CD4+ T cells and yet decreased T cell activation in sepsis. These CD4+ T cell alterations in sepsis are accompanied by widespread loss of cytotoxic cells such as CD8+ cytotoxic cells, NK cells and γδ T cells. Finally, we show reduced levels of B cells, functional defects in the remaining B cells together with immunoglobulin aberrations in sepsis. These insights may inform new therapeutic targets for treating immune impairment in sepsis, including the use of IL-7 or IFN- γ therapy or PD-1 targeting antibodies, which have been shown to reverse T cell dysfunction in murine models of sepsis and a small number of human case studies (92–94). In addition, the alterations in the T cell compartments seen in this study, may aid in stratifying patients for such treatments. Likewise, the B cell impairment evident may be addressed through immunoglobulin supplementation (95). Nonetheless, such findings will need further validation in larger cohorts, however, they highlight the potential of combining single-cell multi-omics with clinically relevant phenotyping to refine treatment and prognostic approaches in sepsis.

## Data Availability

The datasets presented in this study can be found in online repositories. The names of the repository/repositories and accession number(s) can be found below: PRJEB96265 (European Nucleotide Archive (ENA); https://www.ebi.ac.uk/ena/browser/view/PRJEB96265).
