# Supplementary material for: Multi-omic analysis of PBMCs in sepsis reveals widespread cytotoxic dysfunction and an increased population of CD69 expressing naïve CD4+ T cells
Source: Front Immunol. 2025 Oct 24;16:1667186. doi: 10.3389/fimmu.2025.1667186 (PMC12591961; doi:10.3389/fimmu.2025.1667186)
Supplement: Supplementary file 1 [file DataSheet1.docx]

**Supplementary information**

**Ethics statement**

Ethical approval for this study was granted by the St. James’s Hospital Research and Ethics Committee (Ethic approval code 2015-03). Informed written consent was obtained from participants. When this was not possible, assent was obtained from the next of kin, as was permitted by the National Consent Advisory Group, for the ethical conduct of research at the time the samples were collected.

**Processing and storage of PBMCs**

Sepsis and bacteraemia PBMCs were isolated from EDTA anticoagulated blood samples by density gradient centrifugation over Lymphoprep (StemCell Technologies; Cat: #07851) and cryopreserved. ‘Healthy’ PBMCs were obtained using SepMate tubes (StemCell Technologies; Cat: #15450) as per manufacturer’s instructions using Lymphoprep and cryopreserved. Upon recovery, the cell pellets were washed twice in 50 mL PBS (Merck; Cat. #D8537), and the final pellet was re-suspended and stored on ice.

**BD Rhapsody^TM^ single-cell sequencing pipeline**

This study consisted of a pilot and a study phase using retrospective PBMC samples from people with bacteraemia (n=3) and sepsis (n=14). The control ‘healthy’ PBMC samples were freshly collected. The pilot phase consisted of two samples multiplexed per four cartridges for a total of eight samples (control = 2, sepsis = 3, bacteraemia = 3). The study phase consisted of three samples multiplexed across six cartridges for a total of thirteen samples (same control per cartridge = 1, bacteraemia = 1 (repeat from pilot study), sepsis = 11). This gave a total of three bacteraemia, fourteen sepsis and three control samples. A total of 46 AbSeq Ab-Oligos were used in the pilot study (Supplementary Table S2) and 47 in the study phase (addition of CD64, BD; Cat. #940023), however, as CD64 was not detected this was removed from the analysis. This targeted panel was designed to enable profiling of most cell subpopulations present within PBMCs, and to include markers of activation, differentiation, homing, and exhaustion. Similarly, many of the selected markers are altered in other infectious and inflammatory diseases. Several modifications to the manufacturer’s protocol were made over the course of the study in relation to preparing the cells for loading onto the cartridge, due to the poor viability of samples pre and post recovery. These alterations are provided below and included switching from sequential to co-labelling, altering incubation time, increasing cell capture rate, and flow sorting cells prior to loading onto cartridge.

*Preparation of samples for cell loading in pilot phase (Refer to Table 1)*

Following PBMC recovery, samples were centrifuged and re-suspended in cold Sample Buffer (BD; BD Rhapsody^TM^ Cartridge Reagent Kit, Cat: #633731) and filtered through a Falcon tube with Cell Strainer Cap (Corning; Cat. #352235). For run 1 the viability of cells was assessed as well as cells counted using 0.005 mM Draq7 (BD; Cat. # 564904) and 0.01 mM Calcein AM (Thermo Fisher; Cat. #C1430), 1 million cells were then centrifuged and treated with 100 µL Fc Block as per manufacturer’s protocol (Fc Block solution – 114 µL Stain Buffer (2% heat inactivated FBS (Merck; Cat. #F7524) and 1 mM EDTA in PBS), and 6 µL Fc Block (BD Pharmingen Human BD Fc Block; Cat. #564219). For run 2-4, cells were sorted on the BD FACS Melody cell sorter (live/dead) prior to Fc Block. Run 1 was sequentially labelled with AbSeq Ab-Oligos and Sample Tags as per manufacturer’s instructions with a 45 min incubation time for both labelling steps. Run 2-4 were co-labelled with AbSeq Ab-Oligos and Sample Tags as per manufacturer’s instructions with a 45 min incubation time for run 2, and 30 min for run 3/4. Washing steps post labelling was a total of three washes for run 1 and two washes for run 2-4.

*Preparation of samples for cell loading in study phase (Refer to Table 2)*

For run A/B, cells were sorted based on live/dead via flow cytometry, centrifuged and entire pellet resuspended in 25 µL Fc Block solution (24 µL Stain Buffer and 6 µL Fc Block). All samples were co-labelled with AbSeq Ab-Oligos and Sample Tags as per manufacturer’s instructions with a 30 min incubation time, with two washes following labelling. Given that cells were subjected to Fc Block and in line with an updated manufacturer’s protocol, a 175 µL mix was prepared containing 2 µL per AbSeq Ab-Oligos with the remaining balance Stain buffer. This solution was added directly to the appropriate Sample Tag tube as per manufacturer’s instructions. Two washes were completed following incubation. For run C-F, cells were sorted on live/dead via flow cytometry following co-labelling and sorted directly into cold Sample Buffer (BD; BD Rhapsody^TM^ Cartridge Reagent Kit, Cat: #633731), with volume adjusted as needed for cartridge loading (total volume 585 µL). Following this, 3.1 µL Calcein AM (Thermo Fisher; Cat. #C1430) was added, the sample incubated at room temperature for 5 minutes and cells loaded onto cartridge as per manufacturer’s protocol.

*Library preparation*

Cycling conditions were dependent upon initial cell number captured on the BD Rhapsody^TM^ system (as per manufacturer’s instructions). Products were purified as recommended with the Agencourt AMPure XP magnetic beads (Beckman Coulter; Cat. #A63880), and assayed using the Qubit^TM^ dsDNA HS Assay kit (Thermo Fisher Scientific; Cat. #Q32851) and the Agilent Bioanalyzer with High Sensitivity kit (Agilent; Cat. #5067-4626) following standard protocols. QC of final sequencing libraries was performed using Qubit and the Agilent Bioanalyzer.

*Sequencing*

Based on the initial number of cartridges and Sample Tags used, the index PCR used to prepare final libraries utilised Library Reverse primers 1-4 as outlined in the manufacturer’s protocol with the addition of 10 µM TruSeq D703 (5’- CAAGCAGAAGACGGCATACGAGATAATGAGCGGTGACTGGAGTTCAGACGTGTGCTCTTCCGATC*T) and D704 (CAAGCAGAAGACGGCATACGAGATGGAATCTCGTGACTGGAGTTCAGACGTGTGCTCTTCCGATC*T) as required (Integrated DNA Technologies). Cycling conditions for PCR1, PCR2 and index PCR followed manufacturer’s instructions. Based on Bionazlyer data (Agilent; Cat. #5067-4626) several samples underwent repeat AMPure bead purification (Beckman Coulter; Cat. #A63880) and two samples underwent further purification using a QIAquick Gel Extraction Kit (Qiagen, Cat. #28704). The pilot and study phase cartridge samples were sequenced over two sequencing runs. Final library concentrations were prepared based on the sequencing calculator from BD (BD Targeted Library Sequencing Protocol), which took into account values such as qubit concentration, average library size from Bioanalyzer, cell number captured and reads needed. This calculator provided number of reads/cell desired as well as number of total reads required. The final pooled library concentrations were 4 nM, which was diluted to 2.5 nM for the pilot phase and 2.25 nM for the study phase (spiked with 20% PhiX (Illumina; Cat. #FC-110-3001)), according to Illumina’s instructions (NovaSeq 6000, Denature and Dilute Libraries Guide). Sequencing runs were prepared and performed as per manufacturer’s instructions in the TrinSeq facility utilising v1.5 NovaSeq flowcells (Illumina; NovaSeq 6000 S1 Reagent Kit v1.5 (100 cycles), Cat. #20028319) on a NovaSeq 6000 (Illumina), which allowed for paired end reads (2 x 75 bp).

*BD Rhapsody™ pipeline*

Briefly, read pairs that displayed a low sequencing quality were removed. R1 reads were then analysed to identify the cell label section (CLS), common sequences (L), Unique Molecular Identifier (UMI) sequence, and poly(T) tail. The R2 reads were assessed by alignment with a transcript sequence in the reference using Bowtie2. The remaining R1 and R2 read pairs were collapsed into a single raw molecule that contained the same UMI sequences. These raw counts underwent UMI adjustment algorithms using the BD Biosciences tool. UMI errors were identified and adjusted using recursive substitution error correction (RSEC), thus removing artifact molecules. Using the BD Single-Cell Multiplexing Kit, barcoded oligo-nucleotide antibodies (Supplementary Table S2) were used to identify the sample of origin and identify high quality singlets and remove multiplets.

*Cell clustering*

Prior to clustering, the data was integrated using the reciprocal principal component analysis pipeline. Subsequent dimensionality reduction and clustering was performed. For T cell analysis, contaminating B cells and clusters believed to be apoptotic due to expression of contradictory cell type lineage Abseq markers, high expression of all Abseq markers and low RNA content (i.e., CD3+ T cells expressing monocyte-specific markers CD163) with a cutoff of < 30,000 Abseq counts. For T cell and NK cell analysis, clustering resolution was set to 0.8, wherein stable clustering was observed. For B cell analysis, a clustering resolution of 0.2 was used. Clusters were annotated manually using known cell type markers and their corresponding Abseqs and genes (Supplementary Table S4) and the FindMarkers and FindConservedMarkers functions implemented in Seurat V4. Differentially expressed genes and proteins were identified using MAST in Seurat V4.0 (1).

**Table 1: Pilot phase samples**

| **Run #** | **Sample mix** | **Cell capture aim** | **# of wells with viable cell at load** | **Captured (bead with viable cell)** | **Cell multiplet rate** |
| --- | --- | --- | --- | --- | --- |
| 1 | S/B | 24,000 | 4086 | 2901 | 0.50% |
| 2 | S/C | 25,000 | 14393 | 12151 | 3.10% |
| 3 | S/B | 30,000 | 7945 | 6751 | 2.00% |
| 4 | B/C | 30,000 | 15211 | 12716 | 3.20% |

S = sepsis, B = bacteraemia, C = control

**Table 2: Study phase samples**

| **Run #** | **Sample mix** | **Cell capture aim** | **# of wells with viable cell at load** | **Captured (bead with viable cell)** | **Cell multiplet rate** |
| --- | --- | --- | --- | --- | --- |
| A | S/S/C | 29,000 | 2367 | 2039 | 0.30% |
| B | S/S/C | 29,000 | 1923 | 1679 | 0.70% |
| C | S/S/C | 20,000 | 11793 | 10933 | 2.30% |
| D | S/S/C | 22,000 | 13195 | 11183 | 2.60% |
| E | S/S/C | 31,000 | 13683 | 12454 | 2.40% |
| F | S/B/C | 11,000 | 6079 | 5593 | 1.10% |

S = sepsis, B = bacteraemia, C = control

1. Finak G, McDavid A, Yajima M, Deng J, Gersuk V, Shalek AK, et al. MAST: a flexible statistical framework for assessing transcriptional changes and characterizing heterogeneity in single-cell RNA sequencing data. Genome Biol. 2015;16(1):278.
