# Supplementary material for: Multi-omic analysis of PBMCs in sepsis reveals widespread cytotoxic dysfunction and an increased population of CD69 expressing naïve CD4+ T cells
Source: Front Immunol. 2025 Oct 24;16:1667186. doi: 10.3389/fimmu.2025.1667186 (PMC12591961; doi:10.3389/fimmu.2025.1667186)

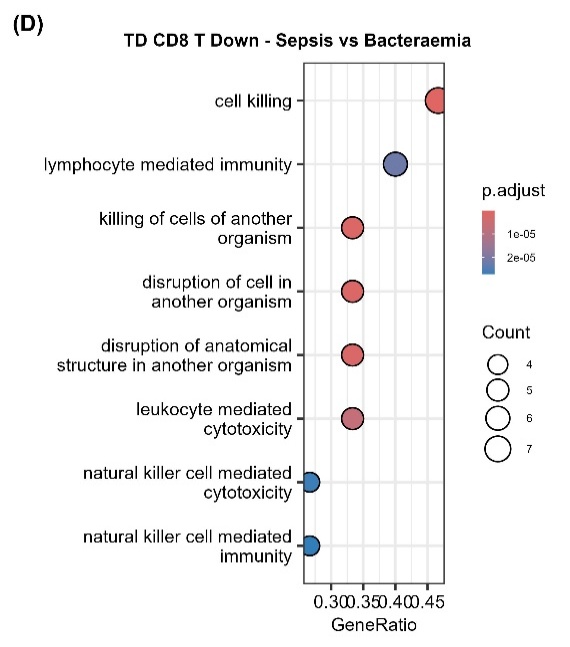

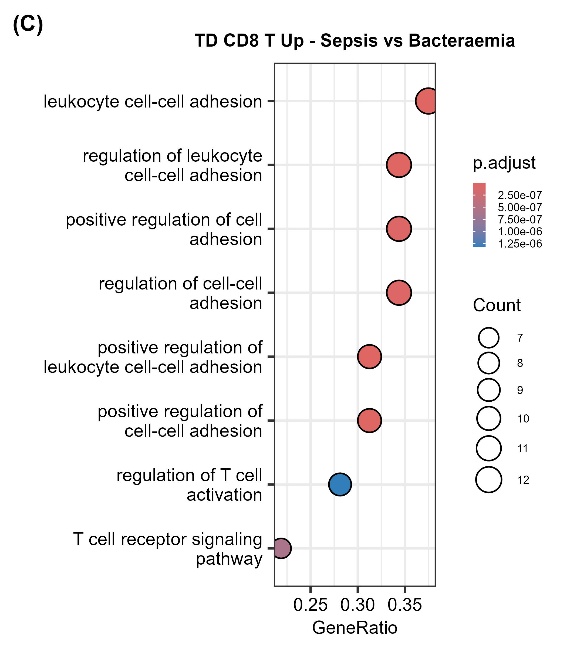

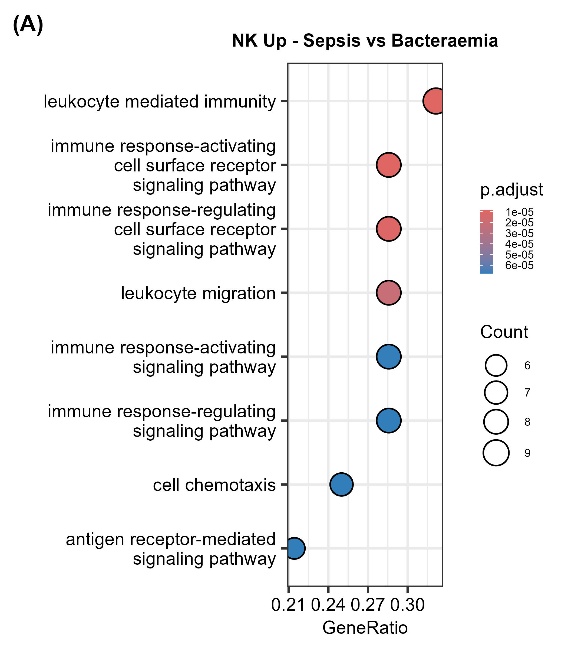

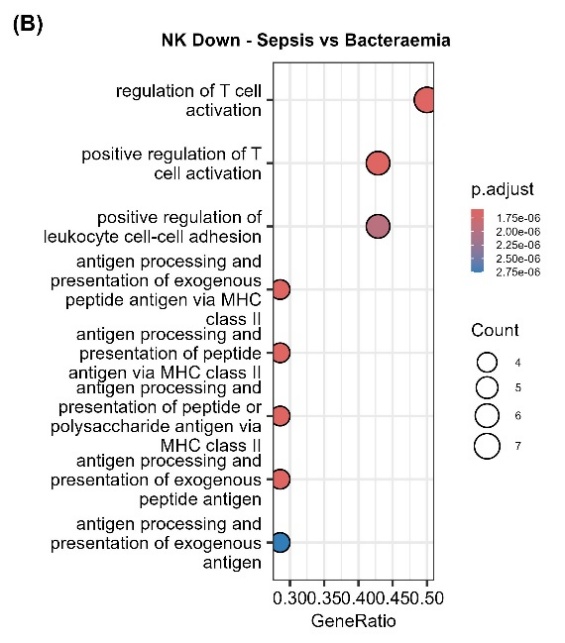

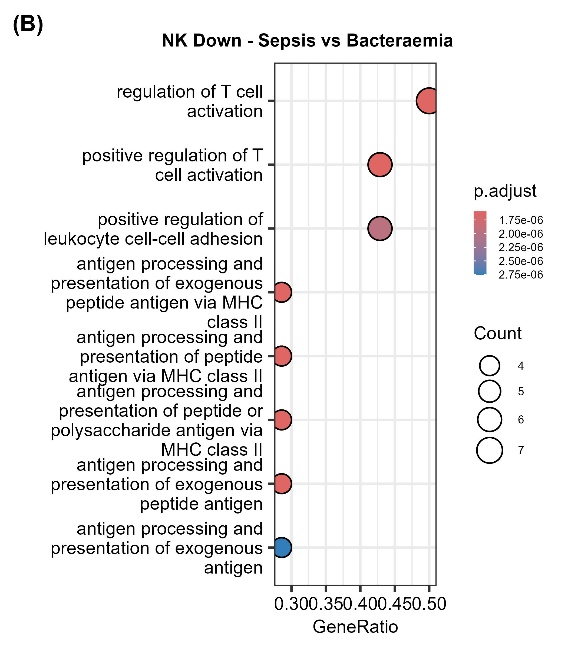

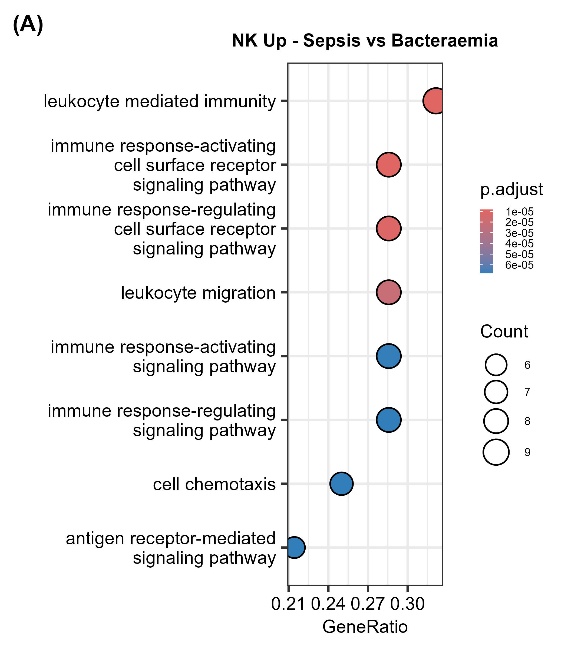

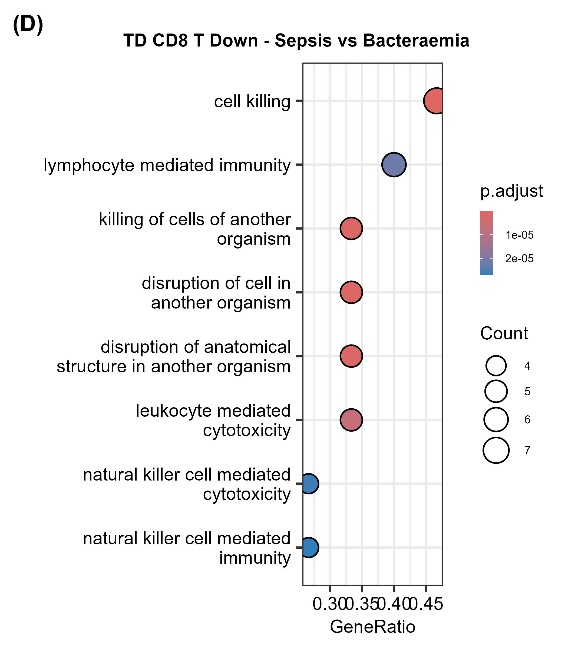

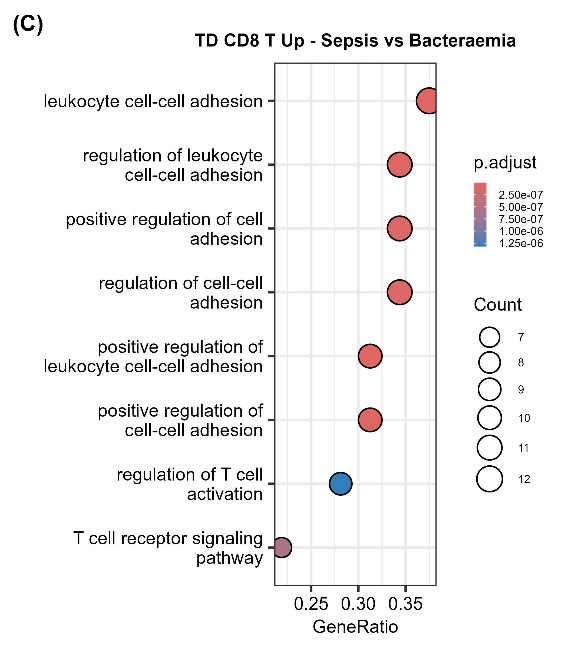


**Supplementary Figure S1**. Dotplots displaying Gene Ontology (GO) Biological Processes (BP) identified that are (A) downregulated in sepsis NK cells compared to bacteraemia NK cells, (B) upregulated in sepsis NK cells compared to bacteraemia NK cells, (C) downregulated in sepsis TD CD8+ T cells compared to bacteraemia TD CD8+ T cells and (D) upregulated in sepsis TD CD8+ T cells compared to bacteraemia TD CD8+ T cells.


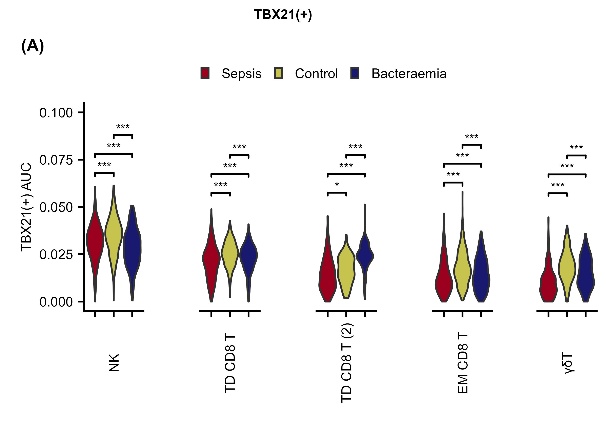

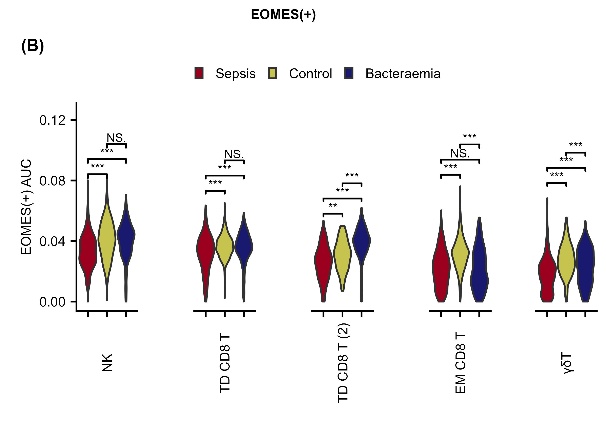


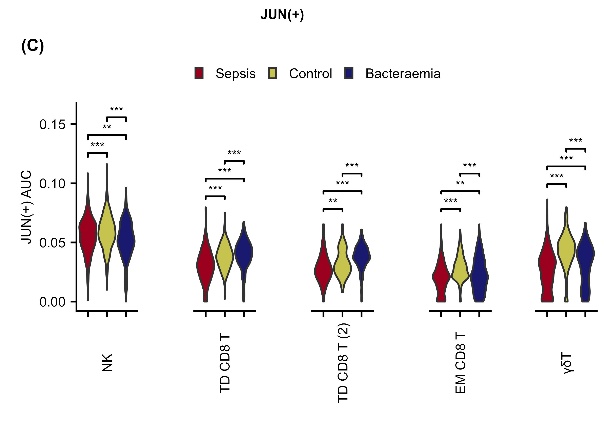

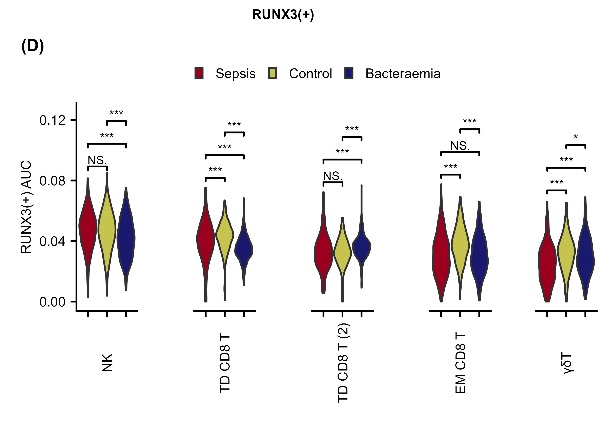


**Supplementary Figure S2**. Violin plots comparing the regulon activity of (A) *TBX21*, (B) *EOMES*, (C) *JUN*, and (D) *RUNX3* in cytotoxic cell subsets between sepsis, bacteraemia, and control samples.

AUC: area under curve


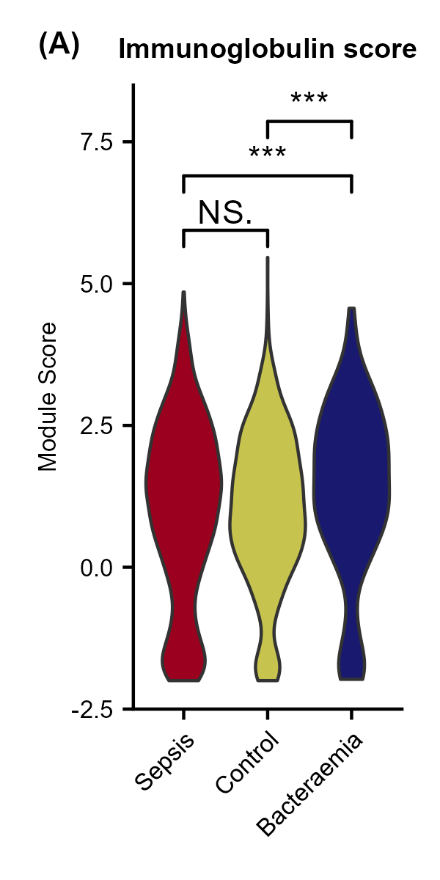

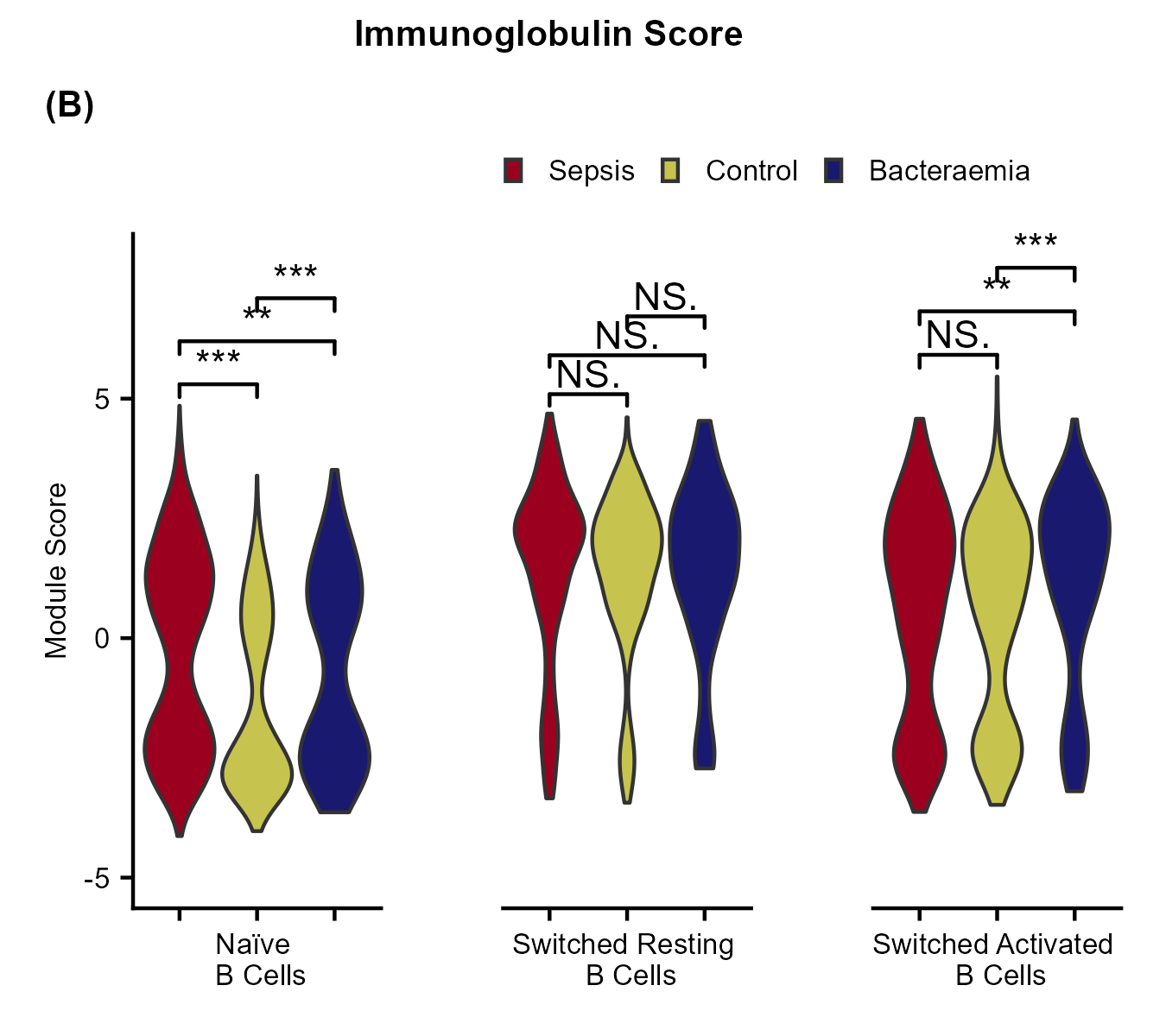

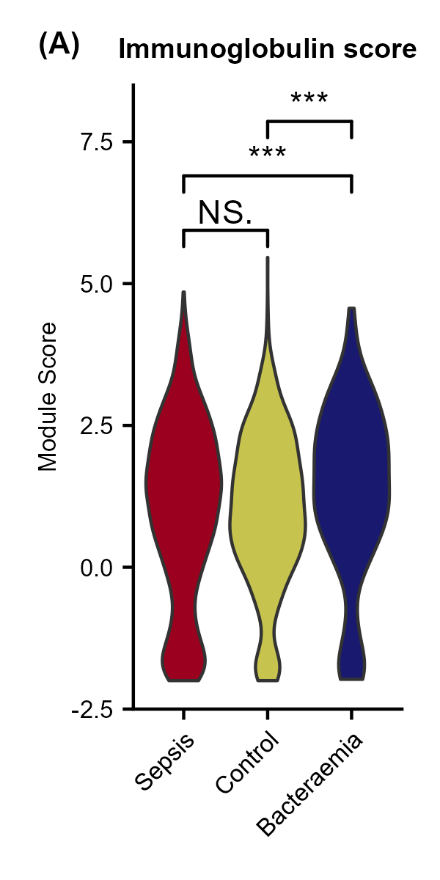

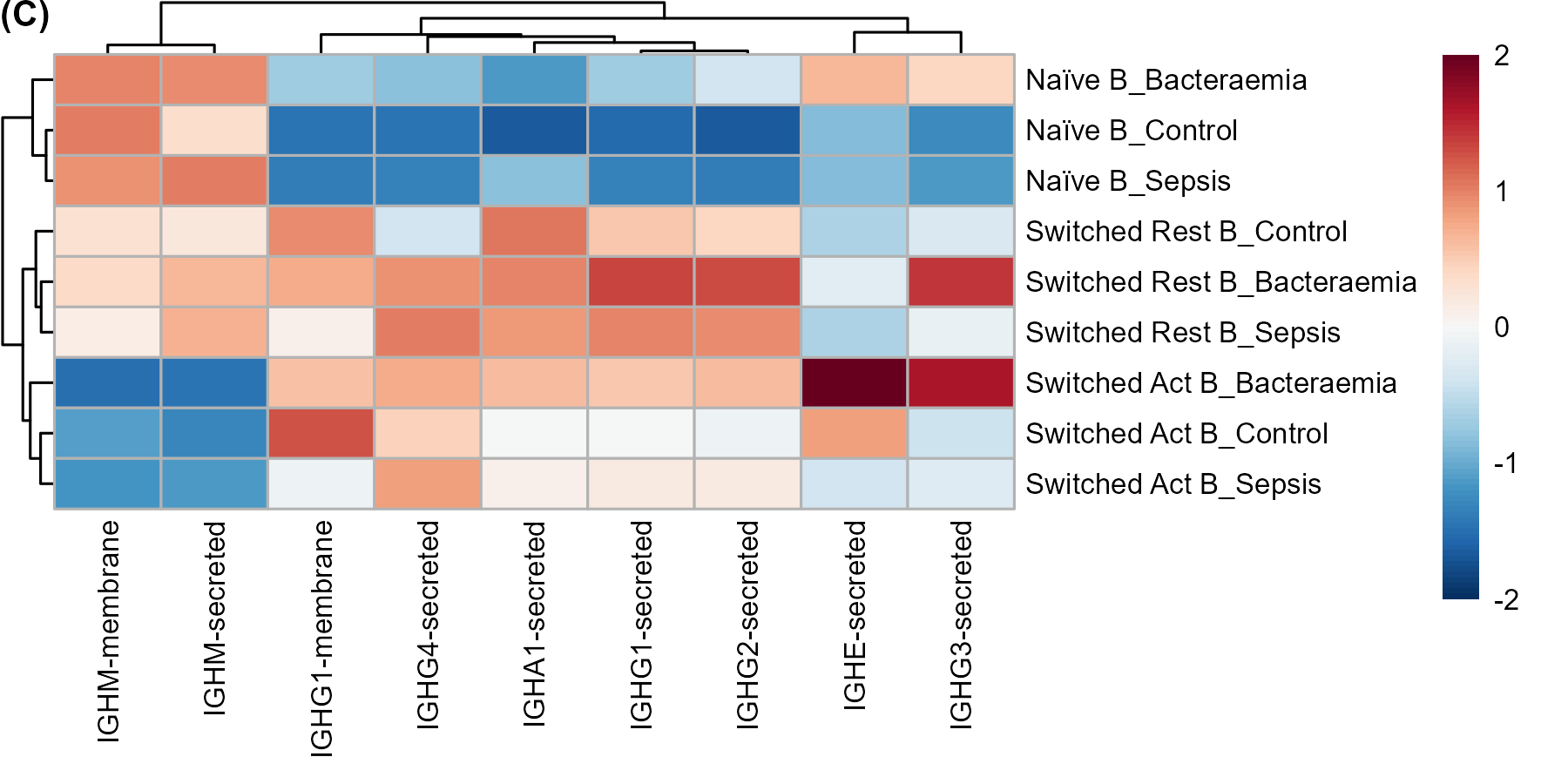


**Supplementary Figure S3**. Violin plots comparing the expression of immunoglobulin genes in (A) total B cells, and (B) B cell subsets in sepsis, bacteraemia and control. (C) Heatmap of immunoglobulin expression.

**Graphical abstract:** Sepsis is responsible for 1 in 5 deaths globally and the majority of those who survive have lasting health issues. A hallmark of sepsis is a deregulated inflammatory response to infection, with leukocytes playing a critical role. This study utilised a targeted single-cell multi-omics approach to characterise peripheral blood mononuclear cell (PBMC) populations and their transcriptomic profiles in an Irish cohort of people with (i) sepsis and (ii)bacteraemia without sepsis. Variable leukocyte distributions were identified, with decreased cytotoxic lymphocytes, including CD8+ T cells, natural killer cells, CD56+ T cells, γδ T cells, mucosal-associated invariant T cells, and increased T helper (Th) cell subsets within sepsis samples. Additionally, PBMCs from sepsis samples displayed an impaired expression profile in effector T cells, resulting in widespread suppression of PBMC cytotoxic activity. These results identify potential mechanisms underlying the functional impairment witnessed in sepsis. Such mechanisms may inform future diagnostic and treatment strategies.

Created in BioRender. Flynn, J. (2025) <https://BioRender.com/10q9yd4>.


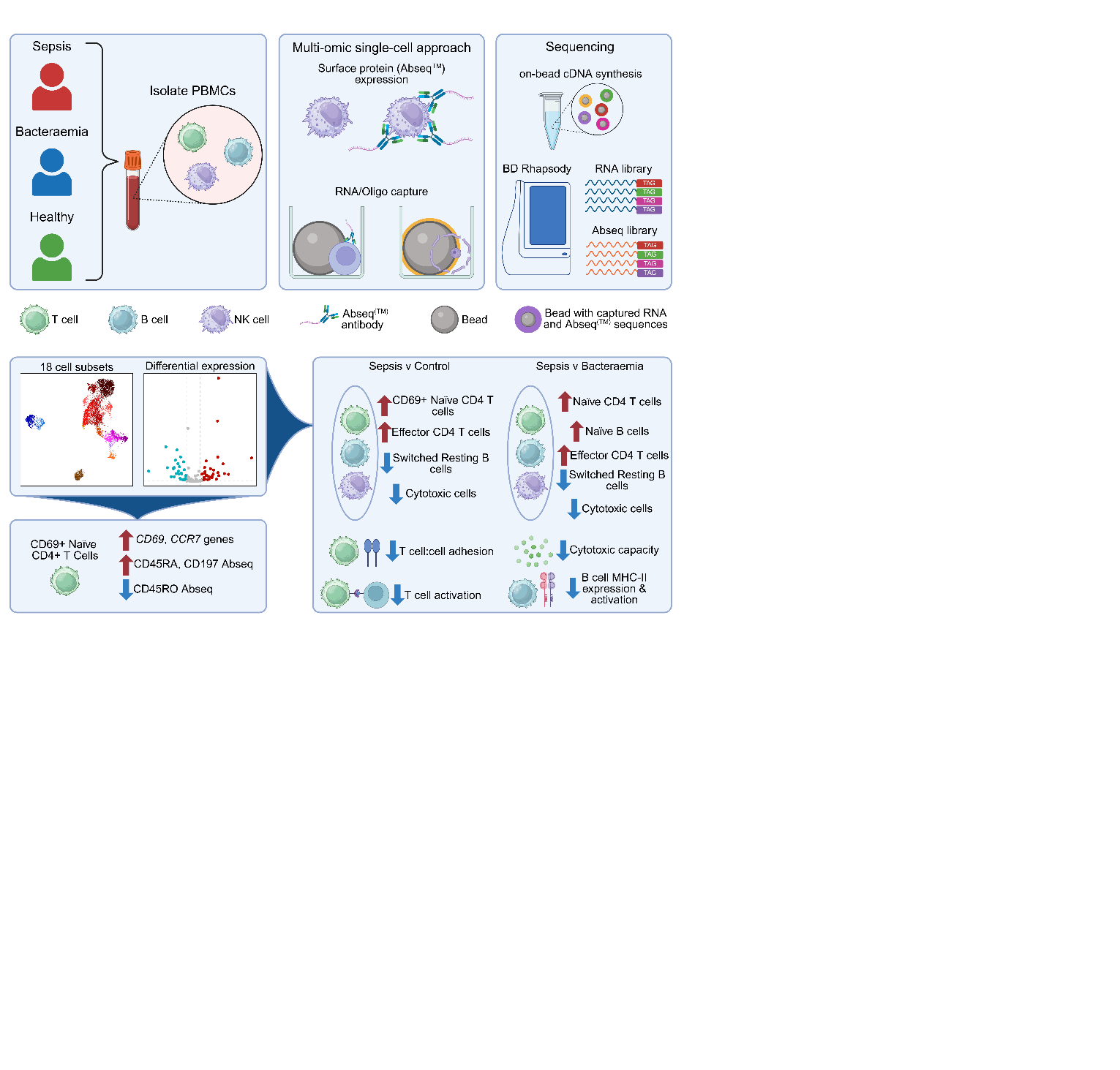

Supplement: Supplementary file 2 [file DataSheet2.docx]
